# Nucleophilic cyclization of *N*-propargylated pyrazole-5-carboxylates: synthesis of pyrazolo[1,5-*a*]pyrazin-4(5*H*)-one derivatives

**DOI:** 10.55730/1300-0527.3790

**Published:** 2026-01-20

**Authors:** Melik Fırat MENGEŞ, Meltem TAN UYGUN

**Affiliations:** Department of Pharmaceutical Chemistry, Faculty of Pharmacy, Van Yüzüncü Yıl University, Van, Turkiye

**Keywords:** Nucleophilic cyclization, pyrazolopyrazinones, fused heterocycles, hydrazine hydrate, pyrazolotriazepinones

## Abstract

Cyclization reactions are among the most important and widely used methods for synthesizing biologically active fused heterocyclic compounds. In the present study, pyrazolopyrazinone derivatives (10a–i) were synthesized from *N*-propargylated pyrazoles via nucleophilic cyclization with hydrazine hydrate under a nitrogen atmosphere. Nuclear magnetic resonance (NMR) spectral analyses confirmed that ring closure proceeded in a manner consistent with the formation of a six-membered pyrazinone moiety. In addition, the pyrazolotriazepinone derivative 15c was synthesized using an alternative approach. In this method, substitution with chloroacetone was first carried out, followed by ring closure with hydrazine hydrate, affording a seven-membered triazepinone system. Comparison of the NMR spectra of the resulting compounds clearly revealed structural differences between the two systems.

## 1. Introduction

Pyrazole derivatives are known for diverse biological activities, and fused pyrazole derivatives—such as pyrazolopyrimidines, pyrazolopyridines, and pyrazolooxazinones—have attracted significant attention for the development of novel therapeutic agents [1,2]. Notably, a heterocyclic compound bearing the pyrazolo[1,5-*a*]pyrazin-4(5*H*)-one scaffold has been identified as an HIV-1 integrase inhibitor (compound 1). Various pyrazolopyrazinones have also been reported to show potential for treating hematological disorders (compound 2), whereas a fluorinated derivative has been shown to be potent against dipeptidyl peptidase-IV (compound 3) [3]. Moreover, among compounds containing the pyrazolopyrazine scaffold, compounds 4–7 (Figure) have been reported to exhibit pronounced activity against lung cancer [2,4–6].

Nevertheless, studies on pyrazolopyrazinones remain limited. The synthesis of these compounds is typically achieved via *N*-alkylation of the pyrazole ring or introduction of a carbonyl-containing alkyl moiety, followed by ring closure with an amine or ammonia [1,2,4,7–10]. A novel methodology for synthesizing this scaffold was previously reported, involving propargylation of the pyrazole ring followed by cyclization with amines (compound 7, Figure) [6]. The purpose of this study is to elucidate the cyclization of propargylated pyrazole derivatives with hydrazine monohydrate to obtain new pyrazolopyrazinone derivatives.

**Scheme 1:**
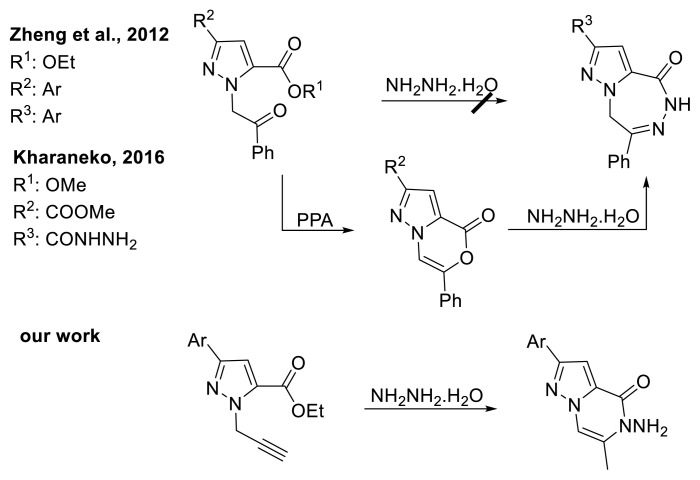
Cyclization of pyrazole derivatives with hydrazine hydrate.

To date, only two reports have examined the reactions of pyrazole derivatives with hydrazine monohydrate [11,12]. In these studies, direct one-step ring closure between *N*-substituted pyrazole derivatives and hydrazine monohydrate could not be achieved. Accordingly, pyrazolooxazinone derivatives were first obtained, and the target compounds were then synthesized in a second step via reaction with hydrazine monohydrate [11,12]. In contrast, the desired compounds (10a–i) were successfully synthesized in a single step via cyclization (Scheme 1).

## 2. Materials and methods

### 2.1. Physical measurements

Commercially available reagents were used without further purification. Nuclear magnetic resonance (NMR) spectra (^1^H and ^13^C) were acquired on an Agilent spectrometer (Agilent Technologies, Santa Clara, CA, USA; 400 MHz for ^1^H and 100 MHz for ^13^C) using tetramethylsilane as an internal reference. Thin-layer chromatography was performed on 0.2-mm silica gel 60 F254 aluminum-backed plates. Liquid chromatography–tandem mass spectrometry (LC–MS/MS) analyses were performed using a Q Exactive mass spectrometer (Thermo Fisher Scientific, Bremen, Germany). Melting points were determined using a Stuart SMP30 melting point apparatus (Cole-Parmer, Stone, Staffordshire, UK). Spectroscopic measurements were performed at the Science Research and Application Center, Van Yüzüncü Yıl University.

### 2.2. Synthesis

#### 2.2.1. General procedure for nucleophilic cyclization of 9a–i with hydrazine monohydrate

The synthesis of the starting compounds (9a–i) has been previously reported in the literature [6]. Hydrazine monohydrate (10 equiv) was added slowly to a solution of ethyl 3-aryl-1-(prop-2-yn-1-yl)-1*H*-pyrazole-5-carboxylate derivatives 9a–i (1.0 equiv) in ethanol (EtOH) (15 mL) under a nitrogen atmosphere. The mixture was heated under reflux for 24 h. After completion of the reaction, EtOH was removed in vacuo. Water (20 mL) was then added. The crude product was extracted with ethyl acetate (3 × 20 mL), and the organic phase was then concentrated in vacuo [13].

Because 11a was the sole product formed, the crude material was purified by column chromatography (ethyl acetate/hexane, 1:2), and the solvents were removed in vacuo to afford a colorless, viscous liquid. Upon standing in chloroform at room temperature for 3 days, 11a was quantitatively converted to 10a [14].

Analysis of the NMR spectra indicated the presence of mixtures of tautomers 10b–d and 11b–d, and their relative proportions were subsequently quantified (see Supporting information, Figures S1–S3). The crude mixtures of 11b–d and 10b–d were allowed to rearrange in chloroform at room temperature for 3 days; the mixtures were then purified by column chromatography (ethyl acetate/hexane, 1:2), and the solvents were removed in vacuo to afford the corresponding pyrazolopyrazinones 10b–d.

Because the 10e–i derivatives were the sole products formed, the crude products were purified by column chromatography (ethyl acetate/hexane, 1:2), and the solvents were removed in vacuo.

5-amino-6-methylene-2-phenyl-6,7-dihydropyrazolo[1,5-*a*]pyrazin-4(5*H*)-one (11a)

Yield: 96% (0.272 g) (before rearrangement). ^1^H NMR (400 MHz, CDCl_3_) δ 7.81–7.78 (m, 2H, Ph–H), 7.44–7.39 (m, 2H, Ph–H), 7.36–7.32 (m, 1H, Ph–H), 7.16 (s, 1H, Prz–H), 5.34 (dd, *J* = 2.8, 1.5 Hz, 1H, =CH_2_), 5.18 (t, *J* = 1.5 Hz, 2H, NCH_2_), 4.68 (dd, *J* = 2.8, 1.5 Hz, 1H, =CH_2_), 4.60 (s, 2H, NH_2_).

5-amino-6-methyl-2-phenylpyrazolo[1,5-*a*]pyrazin-4(5*H*)-one (10a)

White powder; yield: 96% (0.272 g) (after rearrangement). Mp: 226–228 °C. ^1^H NMR (400 MHz, DMSO-d_6_) δ 7.96 (d, *J* = 8.1 Hz, 2H, Ph–H), 7.74 (br s, 1H, =CH), 7.50 (s, 1H, Prz–H), 7.45 (t, *J* = 7.6 Hz, 2H, Ph–H), 7.36 (t, *J* = 7.1 Hz, 1H, Ph–H), 5.67 (s, 2H, NH_2_), 2.31 (s, 3H, CH_3_). ^13^C NMR (100 MHz, DMSO-d_6_) δ 154.0, 151.2, 133.0, 132.1, 129.2, 128.9, 128.5, 125.8, 107.3, 100.8, 15.9. LC–MS/MS m/z [M + Na]^+^: calcd for C_13_H_12_N_4_O, 263.0903; found, 263.0910.

5-amino-2-(4-fluorophenyl)-6-methylpyrazolo[1,5-*a*]pyrazin-4(5*H*)-one (10b)

White powder; yield: 15% (0.042 g) (before rearrangement). Mp: 204–206 °C. ^1^H NMR (400 MHz, CDCl_3_) δ 7.84 (dd, *J* = 8.4, 5.5 Hz, 2H, Ph–H), 7.31 (br s, 1H, =CH), 7.23 (s, 1H, Prz–H), 7.12 (t, *J* = 8.5 Hz, 2H, Ph–H), 4.71 (br s, 2H, NH_2_), 2.38 (s, 3H, CH_3_). ^13^C NMR (100 MHz, CDCl_3_) δ 163.1 (d, *J* = 247.9 Hz), 155.6, 151.7, 133.2, 128.7, 128.5 (d, *J* = 3.2 Hz), 127.9 (d, *J* = 8.2 Hz), 115.9 (d, *J* = 21.7 Hz), 108.1, 101.3, 16.6. LC–MS/MS m/z [M + Na]^+^: calcd for C_13_H_11_FN_4_O, 281.0809; found, 281.0815.

5-amino-2-(4-chlorophenyl)-6-methylpyrazolo[1,5-*a*]pyrazin-4(5*H*)-one (10c)

White powder; yield: 38% (0.108 g) (before rearrangement). Mp: 263–265 °C. ^1^H NMR (400 MHz, CDCl_3_) δ 7.81 (d, *J* = 8.5 Hz, 2H, Ph–H), 7.41 (d, *J* = 8.5 Hz, 2H, Ph–H), 7.32 (br s, 1H, =CH), 7.27 (s, 1H, Prz–H), 4.73 (br s, 2H, NH_2_), 2.40 (s, 3H, CH_3_). ^13^C NMR (100 MHz, CDCl_3_) δ 155.6, 151.5, 134.5, 133.3, 130.8, 129.2, 128.9, 127.4, 108.0, 101.6, 16.6. LC–MS/MS m/z [M + Na]^+^: calcd for C_13_H_11_ClN_4_O: 297.0514; found, 297.0521.

5-amino-2-(4-bromophenyl)-6-methylpyrazolo[1,5-*a*]pyrazin-4(5*H*)-one (10d)

White powder; yield: 61% (0.175 g) (before rearrangement). Mp: 229–231 °C. ^1^H NMR (400 MHz, DMSO-d_6_) δ 7.93–7.89 (m, 2H, Ph–H), 7.73 (br s, 1H, =CH), 7.66–7.62 (m, 2H, Ph–H), 7.53 (d, *J* = 0.6 Hz, 1H, Prz–H), 5.67 (s, 2H, NH_2_), 2.31 (d, *J* = 1.0 Hz, 3H, CH_3_). ^13^C NMR (100 MHz, DMSO-d_6_) δ 153.9, 150.1, 133.1, 131.9, 131.3, 129.5, 127.8, 121.7, 107.3, 101.0, 15.9. LC–MS/MS m/z [M + H]^+^: calcd for C_13_H_11_BrN_4_O, 319.0189; found, 319.0198.

5-amino-6-methyl-2-(4-(trifluoromethyl)phenyl)pyrazolo[1,5-*a*]pyrazin-4(5*H*)-one (10e)

White powder; yield: 85% (0.243 g). Mp: 202–205 °C. ^1^H NMR (400 MHz, CDCl_3_) δ 8.00 (d, *J* = 8.1 Hz, 2H, Ph–H), 7.69 (d, *J* = 8.1 Hz, 2H, Ph–H), 7.36 (d, *J* = 0.5 Hz, 1H, Prz–H), 7.35–7.33 (m, 1H, =CH), 4.75 (s, 2H, NH_2_), 2.42 (d, *J* = 1.1 Hz, 3H, CH_3_). ^13^C NMR (100 MHz, CDCl_3_) δ 155.6, 151.1, 135.7, 133.4, 130.3, 129.3, 126.4, 126.0, 108.0, 102.2, 16.6. LC–MS/MS m/z [M + Na]^+^: calcd for C_14_H_11_F_3_N_4_O, 331.0777; found, 331.0783.

5-amino-2-(4-methoxyphenyl)-6-methylpyrazolo[1,5-*a*]pyrazin-4(5*H*)-one (10f)

White powder; yield: 90% (0.256 g). Mp: 186–188 °C. ^1^H NMR (400 MHz, CDCl_3_) δ 7.81 (d, *J* = 8.7 Hz, 2H, Ph–H), 7.32 (br s, 1H, =CH), 7.22 (s, 1H, Prz–H), 6.97 (d, *J* = 8.7 Hz, 2H, Ph–H), 4.74 (s, 2H, NH_2_), 3.85 (s, 3H, OCH_3_), 2.39 (s, 3H, CH_3_). ^13^C NMR (100 MHz, CDCl_3_) δ 160.1, 155.7, 152.6, 133.1, 128.2, 127.5, 125.0, 114.3, 108.1, 100.9, 55.5, 16.6. LC–MS/MS m/z [M + Na]^+^: calcd for C_14_H_14_N_4_O_2_, 293.1009; found, 293.1011.

5-amino-6-methyl-2-(p-tolyl)pyrazolo[1,5-*a*]pyrazin-4(5*H*)-one (10g)

White powder; yield: 72% (0.204 g). Mp: 275–277 °C. ^1^H NMR (400 MHz, DMSO-d_6_) δ 7.85 (d, *J* = 8.0 Hz, 2H, Ph–H), 7.73 (br s, 1H, =CH), 7.44 (s, 1H, Prz–H), 7.26 (d, *J* = 8.0 Hz, 2H, Ph–H), 5.67 (s, 2H, NH_2_), 2.33 (s, 3H, CH_3_), 2.30 (s, 3H, CH_3_). ^13^C NMR (100 MHz, DMSO-d_6_) δ 153.9, 151.3, 137.9, 132.9, 129.5, 129.3, 129.0, 125.7, 107.3, 100.4, 21.0, 15.9. LC–MS/MS m/z [M + H]^+^: calcd for C_14_H_14_N_4_O, 255.1240; found, 255.1253.

2-([1,1′-biphenyl]-4-yl)-5-amino-6-methylpyrazolo[1,5-*a*]pyrazin-4(5*H*)-one (10h)

Pale yellow powder; yield: 95% (0.272 g). Mp: 276–278 °C. ^1^H NMR (400 MHz, CDCl_3_) δ 7.96 (d, *J* = 7.9 Hz, 2H, Ar–H), 7.69 (d, *J* = 7.9 Hz, 2H, Ar–H), 7.65 (d, *J* = 7.7 Hz, 2H, Ar–H), 7.48–7.45 (m, 2H, Ar–H), 7.39–7.35 (m, 3H, Ar–H/Prz–H/=CH), 4.76 (br s, 2H, NH_2_), 2.41 (s, 3H, CH_3_). ^13^C NMR (100 MHz, CDCl_3_) δ 155.7, 152.3, 141.4, 140.7, 133.2, 131.2, 129.0, 128.6, 127.7, 127.6, 127.1, 126.6, 108.2, 101.7, 16.6. LC–MS/MS m/z [M + H]^+^: calcd for C_19_H_16_N_4_O, 317.1397; found, 317.1410.

5-amino-6-methyl-2-(naphthalen-2-yl)pyrazolo[1,5-*a*]pyrazin-4(5*H*)-one (10i)

White powder; yield: 70% (0.200 g). Mp: 215–217 °C. ^1^H NMR (400 MHz, CDCl_3_) δ 8.36 (s, 1H, Napt–H), 8.01 (d, *J* = 8.6 Hz, 1H, Napt–H), 7.91 (d, *J* = 8.3 Hz, 2H, Napt–H), 7.86 (d, *J* = 5.7 Hz, 1H, Napt–H), 7.51–7.49 (m, 2H, Napt–H), 7.44 (s, 1H, Prz–H), 7.38 (br s, 1H, =CH), 4.76 (br s, 2H, NH_2_), 2.41 (s, 3H, CH_3_). ^13^C NMR (100 MHz, CDCl_3_) δ 155.7, 152.7, 133.6, 133.5, 133.3, 129.6, 128.7, 128.5, 127.9, 127.4, 126.6, 126.5, 125.1, 124.1, 108.2, 101.9, 16.6. LC–MS/MS m/z [M + H]^+^: calcd for C_17_H_14_N_4_O, 291.1240; found, 291.1254.

#### 2.2.2. Ethyl 3-(4-chlorophenyl)-1-propyl-1H-pyrazole-5-carboxylate (12c)

Hydrazine monohydrate (0.5 mL, 10 equiv) was added under air to a solution of ethyl 3-(4-chlorophenyl)-1-(prop-2-yn-1-yl)-1*H*-pyrazole-5-carboxylate 9c (0.300 g, 1.0 equiv) in EtOH (15 mL). The mixture was heated at reflux for 24 h. After completion of the reaction, EtOH was removed in vacuo. Water (20 mL) was then added. The crude product was extracted with ethyl acetate (3 × 25 mL). The organic phase was concentrated in vacuo and purified by column chromatography (ethyl acetate/hexane, 1:2); the solvents were then removed in vacuo to afford the corresponding derivative [14]. Colorless viscous liquid; yield: 65% (0.197 g). ^1^H NMR (400 MHz, CDCl_3_) δ 7.68–7.65 (m, 2H, Ph–H), 7.53–7.49 (m, 2H, Ph–H), 7.09 (s, 1H, Prz–H), 4.56–4.52 (m, 2H, NCH_2_), 4.36 (q, *J* = 7.1 Hz, 2H, OCH_2_), 1.94–1.85 (m, 2H, CH_2_), 1.40 (t, *J* = 7.1 Hz, 3H, CH_3_), 0.94 (t, *J* = 7.4 Hz, 3H, CH_3_). ^13^C NMR (100 MHz, CDCl_3_) δ 159.9, 149.8, 133.3, 132.8, 128.8, 128.0, 125.7, 108.1, 61.1, 53.6, 24.3, 14.4, 11.2.

#### 2.2.3. 2-(4-chlorophenyl)-5-((4-methoxybenzylidene)amino)-6-methylpyrazolo[1,5-*a*]pyrazin-4(5H)-one (13c)

A solution of pyrazolopyrazinone derivative 10c (50 mg, 1.0 equiv) in EtOH (5 mL) was treated with 4-methoxybenzaldehyde (25 mg, 1.0 equiv). After the addition of a few drops of acetic acid, the mixture was heated at reflux for 8 h. After completion of the reaction, the solvent was removed, affording a white powder. The product was obtained in pure form after washing with cyclohexane [14]. Yield: 96% (68 mg). Mp: 247–249 °C. ^1^H NMR (400 MHz, CDCl_3_) δ 9.02 (s, 1H, N=CH), 7.84–7.82 (m, 4H, Ph–H), 7.41 (d, *J* = 8.0 Hz, 2H, Ph–H), 7.36 (s, 1H, =CH), 7.32 (s, 1H, Prz–H), 6.99 (d, *J* = 8.0 Hz, 2H, Ph–H), 3.89 (s, 3H, OCH_3_), 2.36 (s, 3H, CH_3_). ^13^C NMR (100 MHz, CDCl_3_) δ 165.4, 163.1, 152.8, 151.4, 134.4, 132.1, 130.9, 130.7, 129.1, 128.5, 127.4, 125.6, 114.5, 107.9, 102.7, 55.6, 17.2. LC–MS/MS m/z [M + H]^+^: calcd for C_21_H_17_ClN_4_O_2_, 393.1113; found, 393.1129.

#### 2.2.4. Ethyl 3-(4-chlorophenyl)-1-(2-oxopropyl)-1H-pyrazole-5-carboxylate (14c)

Chloroacetone (0.1 mL, 1.1 equiv) and K_2_CO_3_ (0.200 g, 1.0 equiv) were added to a solution of ethyl 3-(4-chlorophenyl)-1*H*-pyrazole-5-carboxylate 8c (0.300 g, 1.0 equiv) in acetone (20 mL). The mixture was heated at reflux for 24 h. After cooling to room temperature, the solvent was removed in vacuo. The crude product was extracted with ethyl acetate (3 × 10 mL), and the organic phase was then concentrated in vacuo. The product was obtained as a white solid without further purification [14]. Yield: 98% (0.36 g). Mp: 82–83 °C. ^1^H NMR (400 MHz, CDCl_3_) δ 7.72 (d, *J* = 7.8 Hz, 2H, Ph–H), 7.37 (d, *J* = 7.8 Hz, 2H, Ph–H), 7.17 (s, 1H, Prz–H), 5.38 (s, 2H, NCH_2_), 4.32 (q, *J* = 7.1 Hz, 2H, OCH_2_), 2.24 (s, 3H, COCH_3_), 1.37 (t, *J* = 7.1 Hz, 3H, CH_3_). ^13^C NMR (100 MHz, CDCl_3_) δ 201.1, 159.8, 149.8, 134.3, 134.1, 130.8, 129.0, 127.0, 108.3, 61.5, 61.4, 27.1, 14.3. LC–MS/MS m/z [M + H]^+^: calcd for C_15_H_15_ClN_2_O_3_, 307.0844; found, 307.0855.

#### 2.2.5. 2-(4-chlorophenyl)-7-methyl-5,8-dihydro-4H-pyrazolo[5,1-*d*][1,2,5]triazepin-4-one (15c)

Hydrazine monohydrate (0.45 mL, 5 equiv) was added slowly to a solution of ethyl 3-(4-chlorophenyl)-1-(2-oxopropyl)-1*H*-pyrazole-5-carboxylate 14c (0.550 g, 1 equiv) in methanol (MeOH) (10 mL) under a nitrogen atmosphere. The mixture was heated at reflux for 24 h. Water (20 mL) was then added. The solvent was removed in vacuo. The crude product was extracted with ethyl acetate (3 × 20 mL). The organic phase was concentrated in vacuo and purified by column chromatography (ethyl acetate/hexane, 1:5); the solvents were then removed in vacuo to afford the pyrazolotriazepinone derivative as a white solid [13]. Yield: 18% (0.088 g). Mp: 191–193 °C. ^1^H NMR (400 MHz, CDCl_3_) δ 8.79 (s, 1H, NH), 7.71 (d, *J* = 8.4 Hz, 2H, Ar–H), 7.38 (d, *J* = 8.4 Hz, 2H, Ar–H), 7.24 (s, 1H, Prz–H), 4.98 (s, 2H, NCH_2_), 2.31 (s, 3H, CH_3_). ^13^C NMR (100 MHz, CDCl_3_) δ 158.7, 157.5, 150.5, 136.8, 134.4, 130.5, 129.2, 127.0, 108.2, 52.2, 23.7. LC–MS/MS m/z [M + H]^+^: calcd for C_13_H_11_ClN_4_O, 275.0694; found, 275.0685.

## 3. Results and discussion

**Scheme 2:**
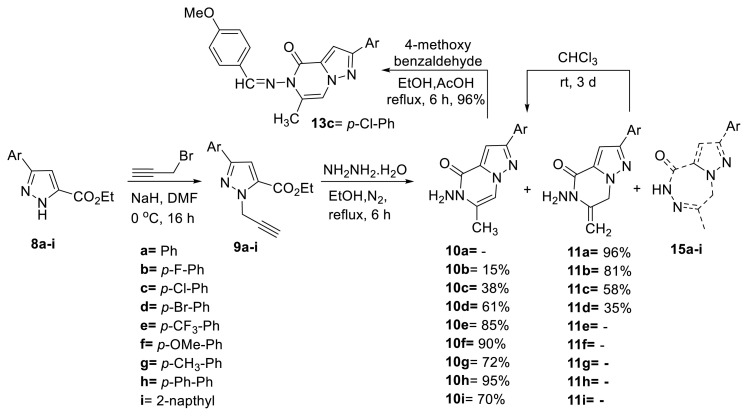
Cyclization of compounds 9a–i and condensation of 10c.

The objective of this research is to generate the target heterocyclic core via intramolecular cyclization of propargyl esters in the presence of hydrazine monohydrate. *N*-propargyl-substituted pyrazole carboxylate derivatives 9a–i were obtained by reacting 3-aryl-1*H*-pyrazole-5-carboxylates 8a–i with propargyl bromide in the presence of NaH as a base (Scheme 2) [6].

**Scheme 3:**
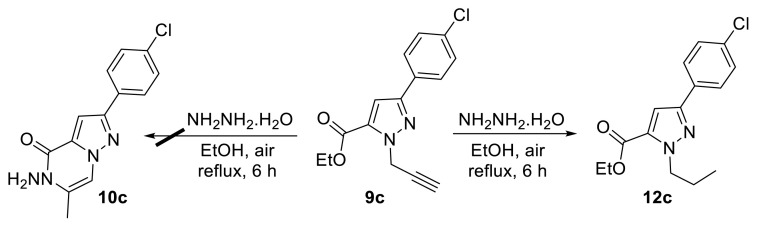
Reaction of propargylated pyrazole ester 9c with hydrazine monohydrate.

The subsequent step involved hydrazine monohydrate-mediated cyclization of propargyl esters 9a–i. For this purpose, compound 9c was reacted with hydrazine monohydrate in EtOH at reflux. However, cyclization did not afford the expected pyrazolopyrazinone structure 10c; instead, compound 12c was formed (Scheme 3) [14].

Menges and Balci proposed that diimide, formed in situ by oxidation of hydrazine hydrate with molecular oxygen, is responsible for hydrogenation of carbon–carbon double and triple bonds [15]. Accordingly, formation of compound 12c can be rationalized by reduction of the propargyl group to a propyl group (see Supporting information, Figure S4). Therefore, the same reaction was repeated under a nitrogen atmosphere [13]. Pyrazolo[1,5-a]pyrazin-4(5*H*)-one 10c and 6,7-dihydropyrazolo[1,5-*a*]pyrazin-4(5*H*)-one 11c were formed in 38% and 58% yields, respectively (Scheme 2).

**Scheme 4:**
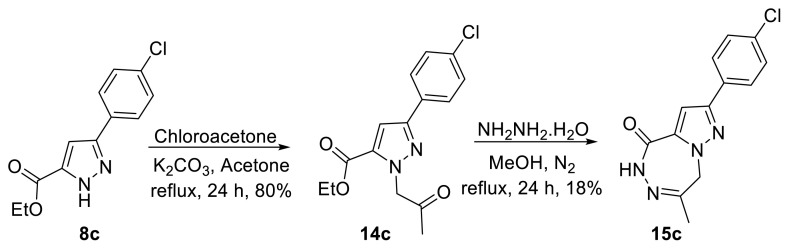
Synthesis of 15c.

The structures of cyclization products 10c and 11c were elucidated based on NMR data. ^1^H NMR analysis of pyrazolopyrazinone derivative 10c indicated the presence of two NH_2_ protons resonating at 4.73 ppm as a broad singlet, and the vinylic proton (=CH) resonating at 7.32 ppm as a broad singlet. Moreover, the CH_3_ protons attached to the double bond resonated at 2.40 ppm as a doublet. Conversely, in 11c, the methylene protons and the two NH_2_ protons appeared at 5.18 and 4.60 ppm as a multiplet and a broad singlet, respectively. Furthermore, the multiplet resonances of the terminal vinylic protons (−C=CH_2_) at 5.36 and 4.70 ppm clearly indicated that the double bond resided outside the ring system. Additionally, tautomerization of the double bond in 11c in chloroform at room temperature led to formation of 10c (Scheme 4). In this study, characterization of this specific tautomer within the series 10a–i was emphasized (see Supporting information, Figures S5–S22).

To obtain additional structural evidence for the NH_2_ group, 10c was reacted with 4-methoxybenzaldehyde in EtOH [14]. The hydrazone 13c was obtained in 96% yield (Scheme 2). Upon disappearance of the NH_2_ proton signals, a resonance attributed to the newly formed N=CH proton was observed at 9.02 ppm, as expected.

In addition to the pyrazolopyrazinone derivative formed from compound 9 upon reaction with hydrazine monohydrate, a pyrazolotriazepinone system (15), potentially obtainable via seven-membered ring closure, was also considered (Scheme 4) [13,14]. Because signals attributable to this system were not detected in the NMR spectra, this structure was excluded. To confirm the structure of 10c, compound 15c was synthesized via an alternative route, and the NMR spectra of pyrazolopyrazinone 10c were compared with those of pyrazolotriazepinone 15c.

Although similar pyrazolotriazepinone systems have been synthesized via two-step procedures employing hydrazine monohydrate, compound 15c has not previously been reported [11,12]. However, Yenice et al. reported synthesis of a pyrrolotriazepinone derivative in a single step under a nitrogen atmosphere using a similar procedure [13]. Based on this precedent, the reaction was performed on the present system to obtain the pyrazolotriazepinone derivative 15c. Treatment of 14c with hydrazine monohydrate under reflux in MeOH afforded the triazepinone derivative 15c in 18% yield (Scheme 4).

^1^H and ^13^C NMR analyses of compounds 10c and 15c exhibited distinct chemical-shift patterns, indicating clear structural differences between the two molecules. In the ^1^H NMR spectrum of 15c, a broad singlet attributed to the NH proton, appeared at 8.79 ppm, whereas the methylene protons exhibited a singlet at 4.98 ppm. Furthermore, in the ^13^C NMR spectrum, signals at 52.2 ppm (methylene carbon) and 23.7 ppm (methyl carbon) supported formation of a seven-membered ring.

**Scheme 5:**
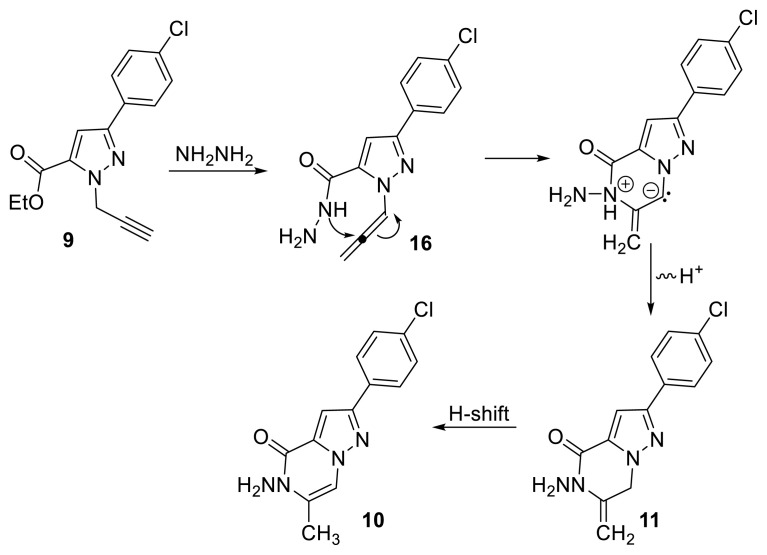
Proposed pathway for nucleophilic cyclization of compound 9.

Based on the experimental results, the proposed pathway for synthesis of compounds 10 and 11 is outlined in Scheme 5. The initial step involved formation of hydrazide 16 from reaction of compound 9 with hydrazine, followed by cyclization. Cyclization was proposed to proceed via formation of an allenic intermediate 16 [14]. Because the central carbon of the allene unit is relatively electron-deficient, it can be attacked by the lone pair on the internal nitrogen, leading to formation of a six-membered ring. Following cyclization, the dihydropyrazolopyrazinone skeleton (11) is formed via proton transfer, whereas the pyrazolopyrazinone moiety (10) is subsequently obtained via a 1,3-H shift.

## 4. Conclusion

In conclusion, a novel and efficient single-step synthetic pathway has been established for the preparation of pyrazolo[1,5-*a*]pyrazin-4(5*H*)-one derivatives via cyclization of propargylated pyrazole derivatives with hydrazine monohydrate. Unlike previous reports, six-membered ring formation via reaction of hydrazine monohydrate with pyrazole esters was achieved for the first time. A mechanistic pathway for synthesis of the compounds was proposed. In addition, a seven-membered triazepinone system was successfully synthesized for the first time via a one-step procedure. Furthermore, the method presented here has been applied to incorporate additional moieties at multiple positions within the target compounds.

## Supplementary Information



## Figures and Tables

**Figure:**
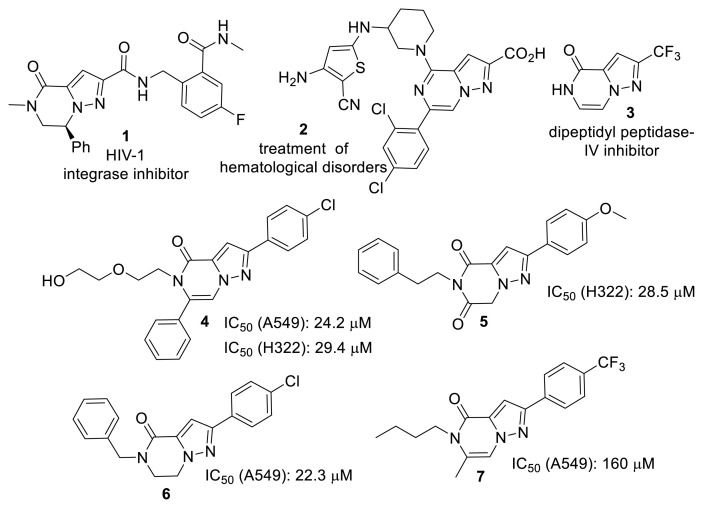
Biologically active pyrazolopyrazinone derivatives.
