# Supplementary material for: Nucleophilic cyclization of N-propargylated pyrazole-5-carboxylates: synthesis of pyrazolo[1,5-a]pyrazin-4(5H)-one derivatives
Source: Turk J Chem. 2026 Jan 20;50(2):199–206. doi: 10.55730/1300-0527.3790 (PMC13189371; doi:10.55730/1300-0527.3790)
Supplement: Supplementary file 1 [file tjc-50-02-199-suppl.pdf]

Nucleophilic cyclization of *N*-propargylated pyrazole carboxylates: Synthesis of pyrazolopyrazinone derivatives

Melik Fırat MENGEŞ[1], Meltem TAN-UYGUN*[1]

[1]Department of Pharmaceutical Chemistry, Faculty of Pharmacy, Van Yüzüncü Yıl University, Van, Turkey

* corresponding author: meltemtan@yyu.edu.tr

Supplementary Material

**Contents**	**Pages**
**1.** ^{1}H & ^{13}C NMR Spectra of Compounds	**S2-S16**
**2.** LC-MS/MS Spectra of Compounds	**S17-S20**

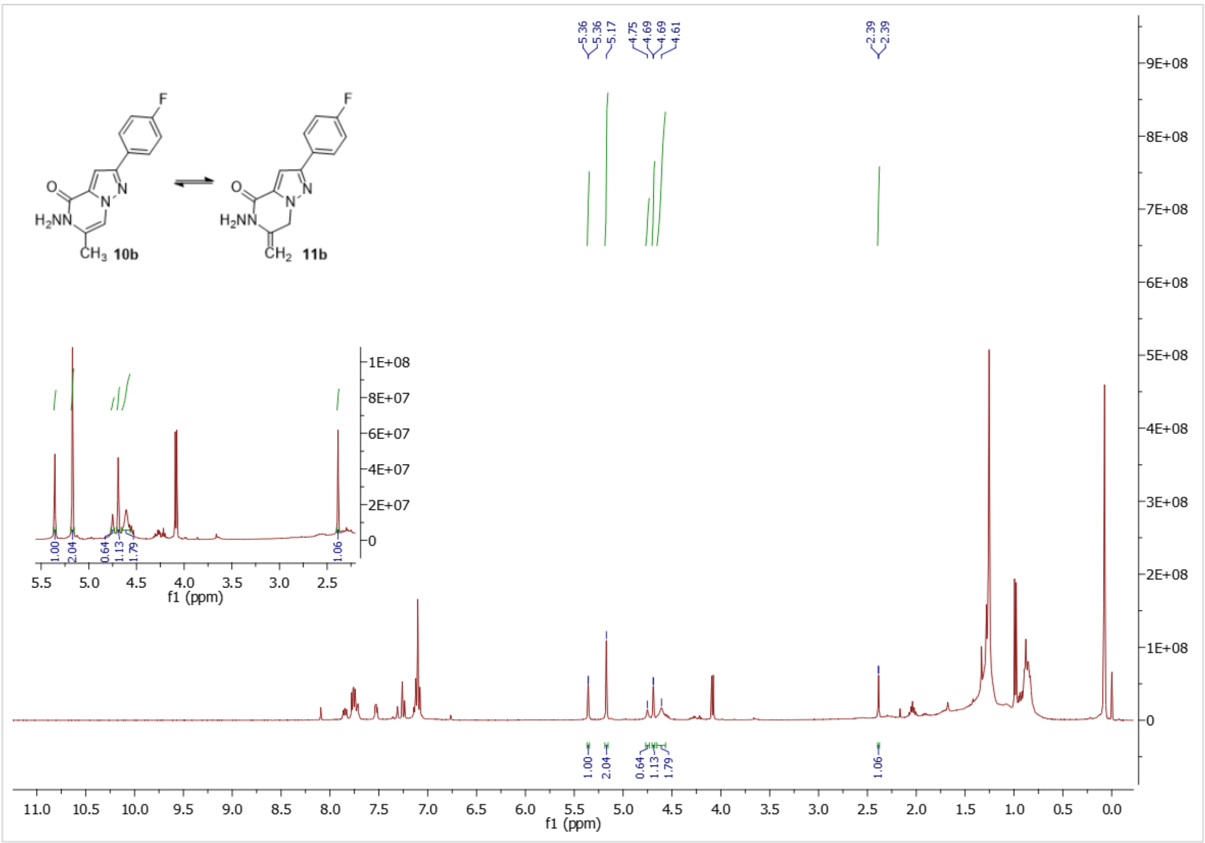

Figure S1. ^{1}H NMR spectrum of compound **10b-11b**

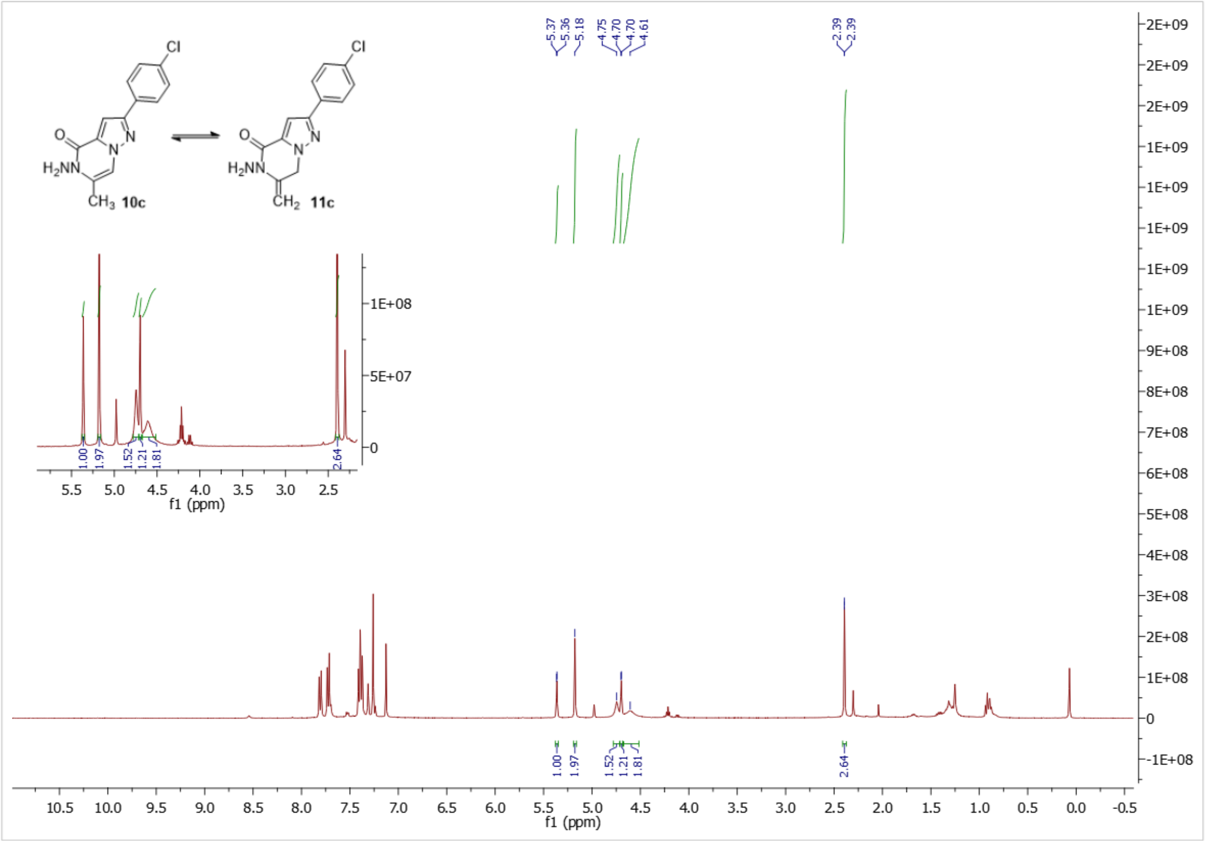

Figure S2. ^{1}H NMR spectrum of compound **10c-11c**

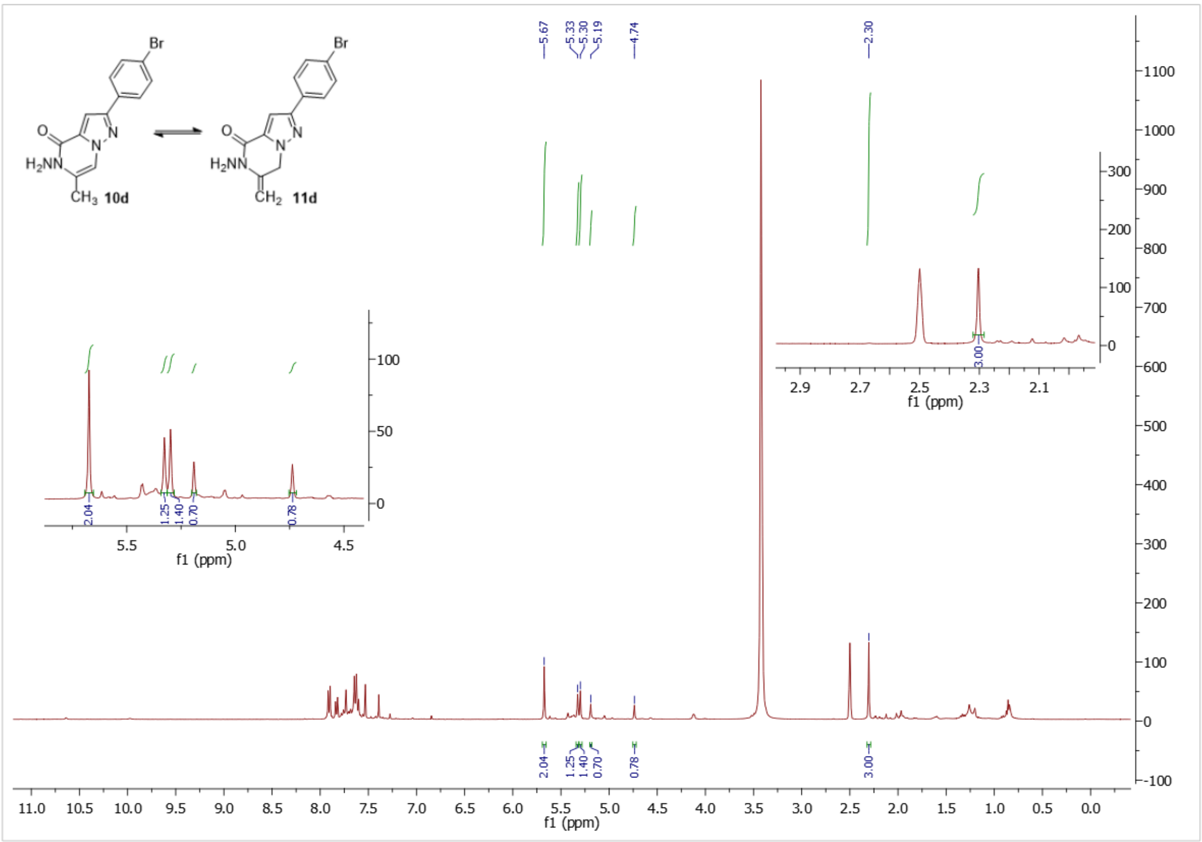

Figure S3. ^{1}H NMR spectrum of compound **10d-11d**

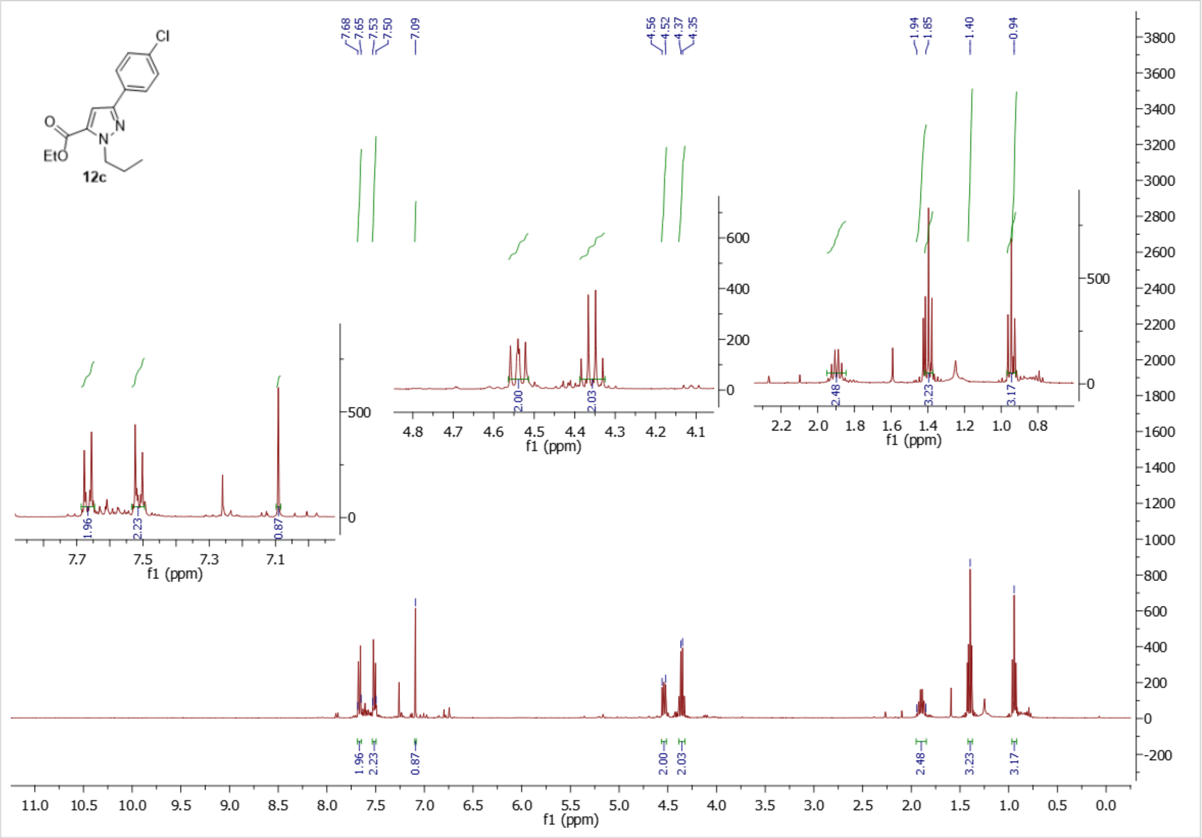

Figure S4. ^{1}H NMR spectrum of compound **12c**

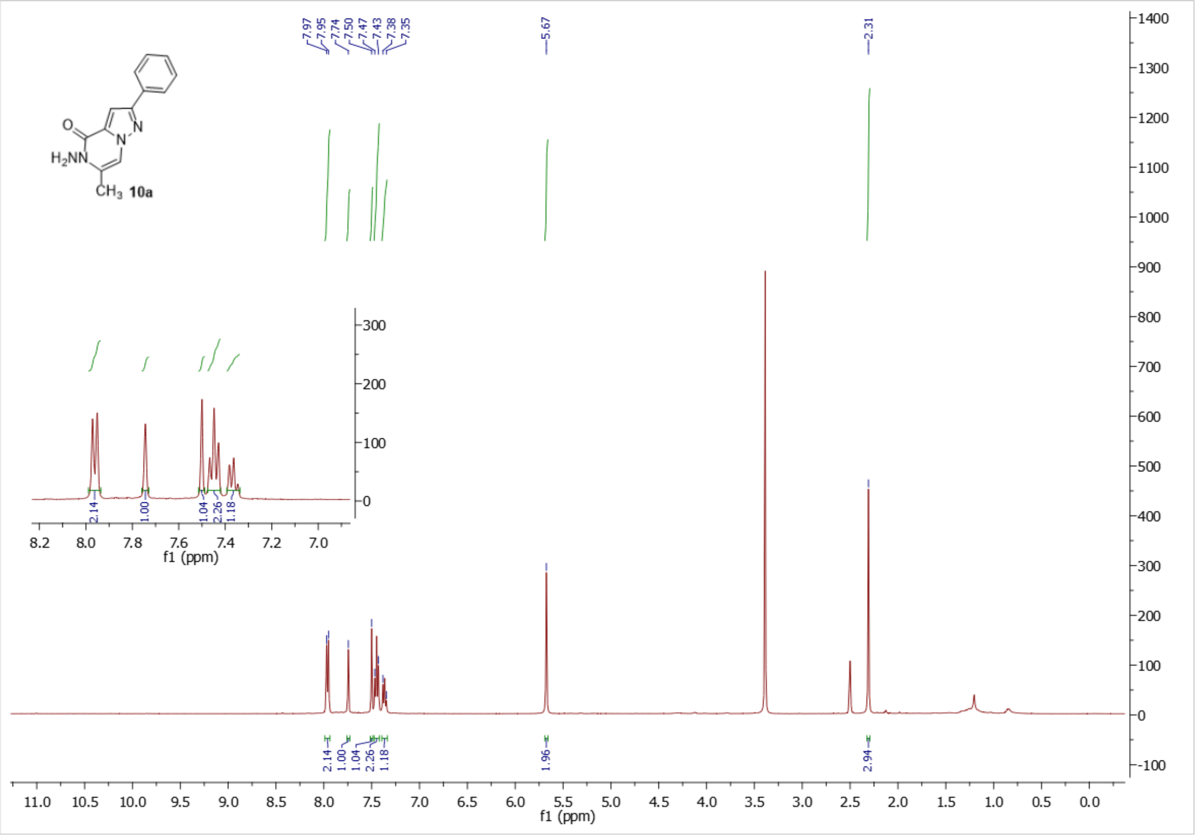

Figure S5. ^{1}H NMR spectrum of compound **10a**

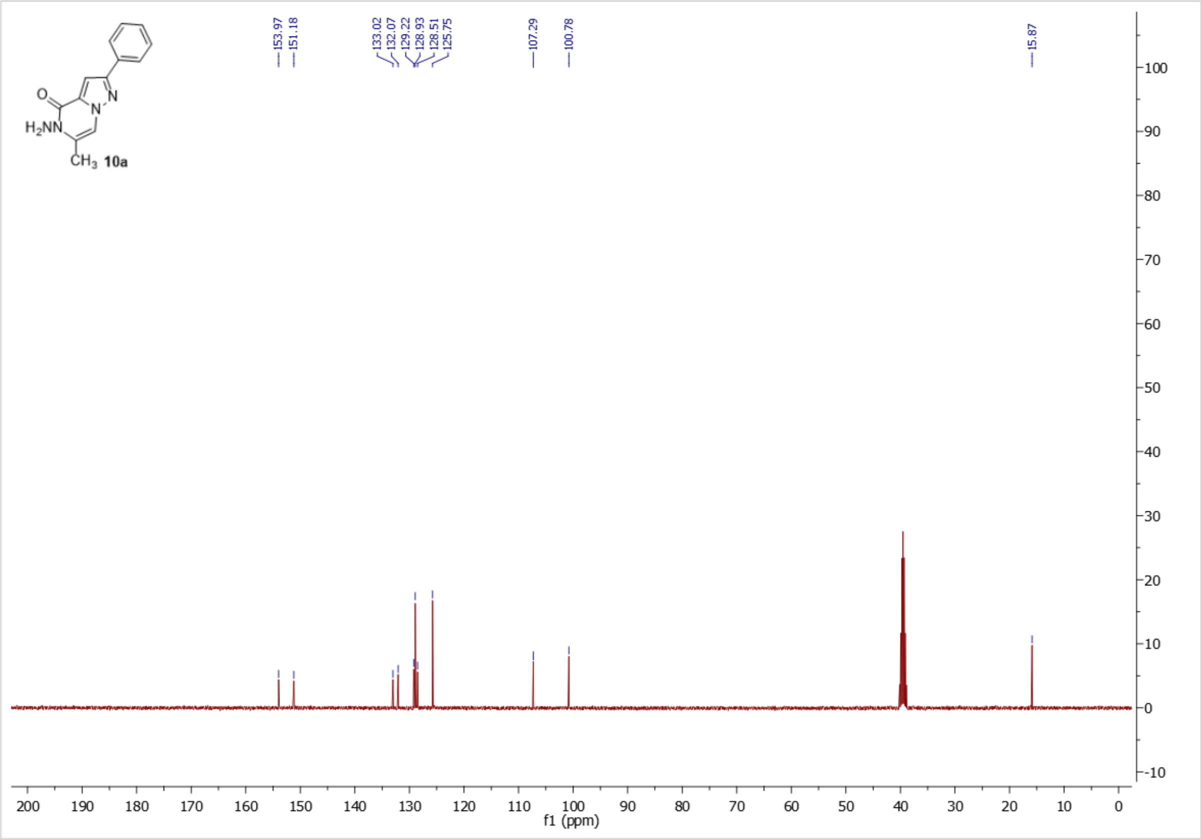

Figure S6. ^{13}C NMR spectrum of compound **10a**

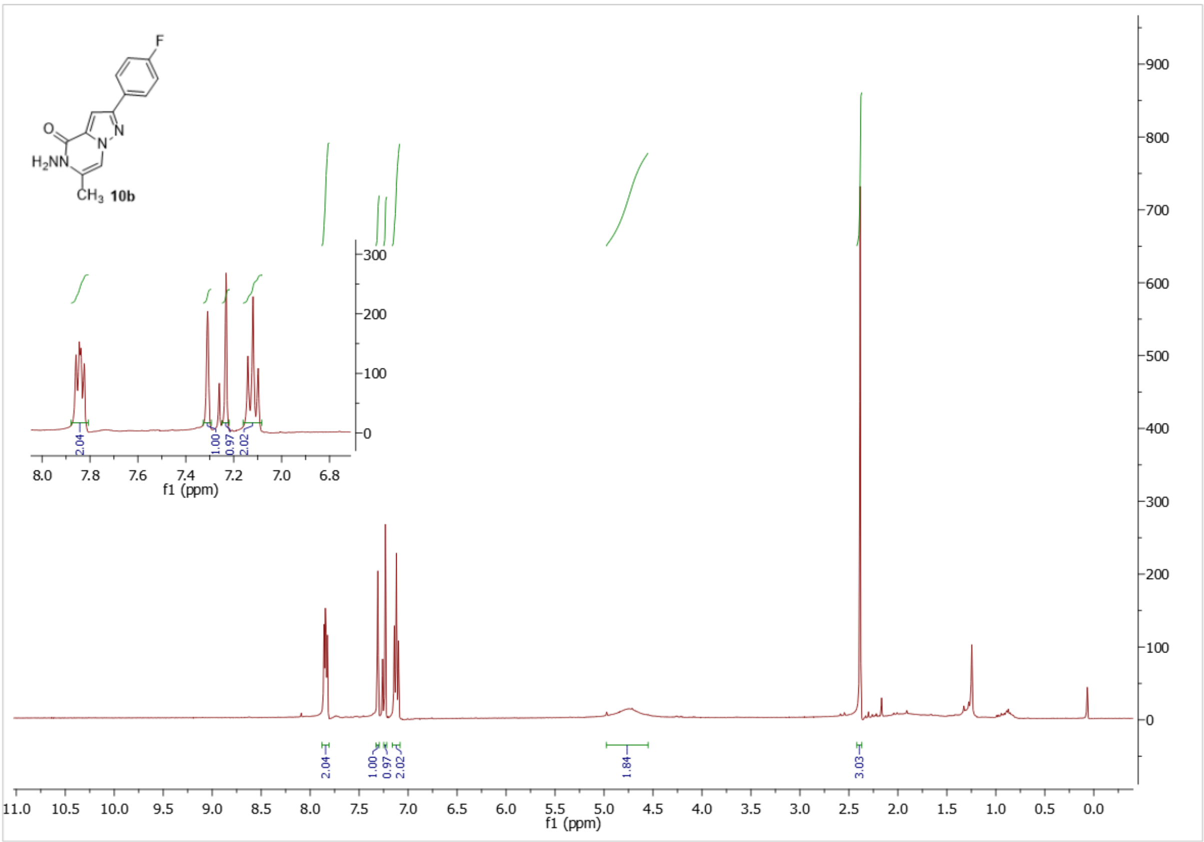

Figure S7. ^{1}H NMR spectrum of compound **10b**

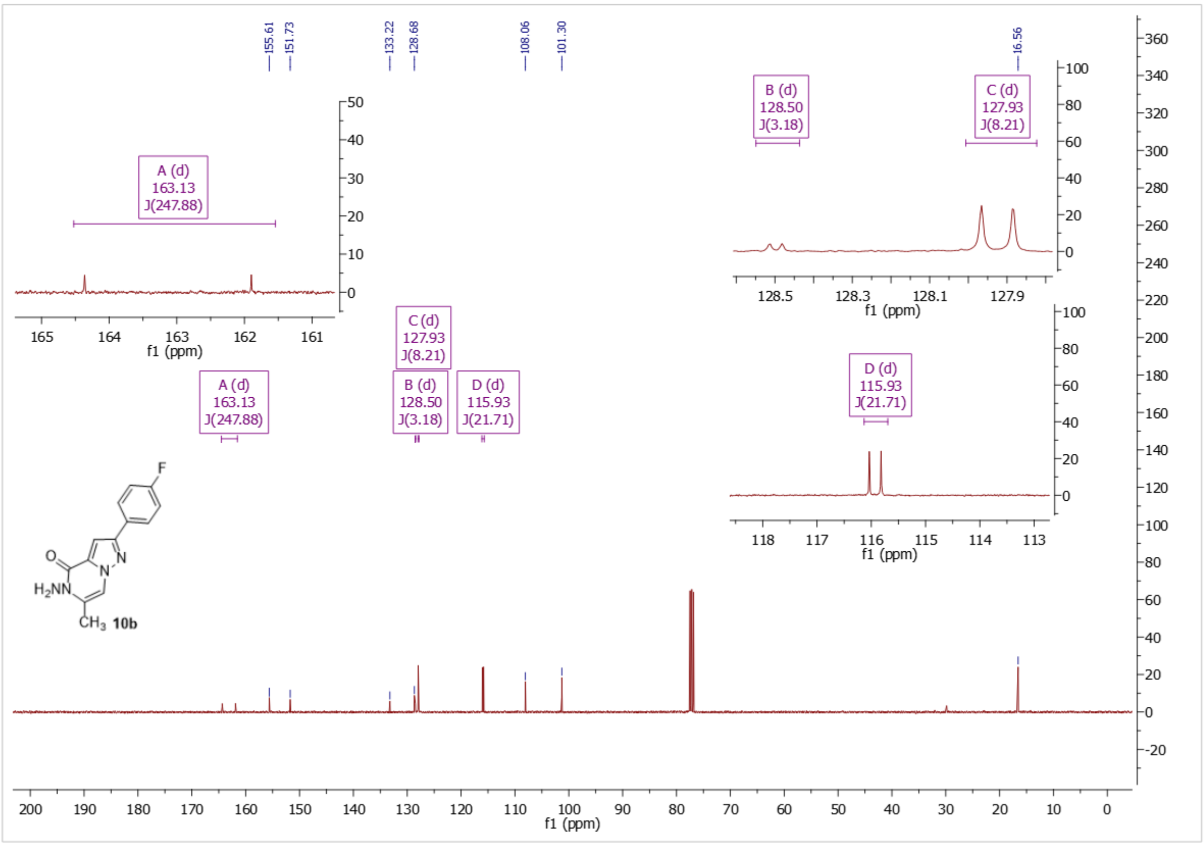

Figure S8. ^{13}C NMR spectrum of compound **10b**

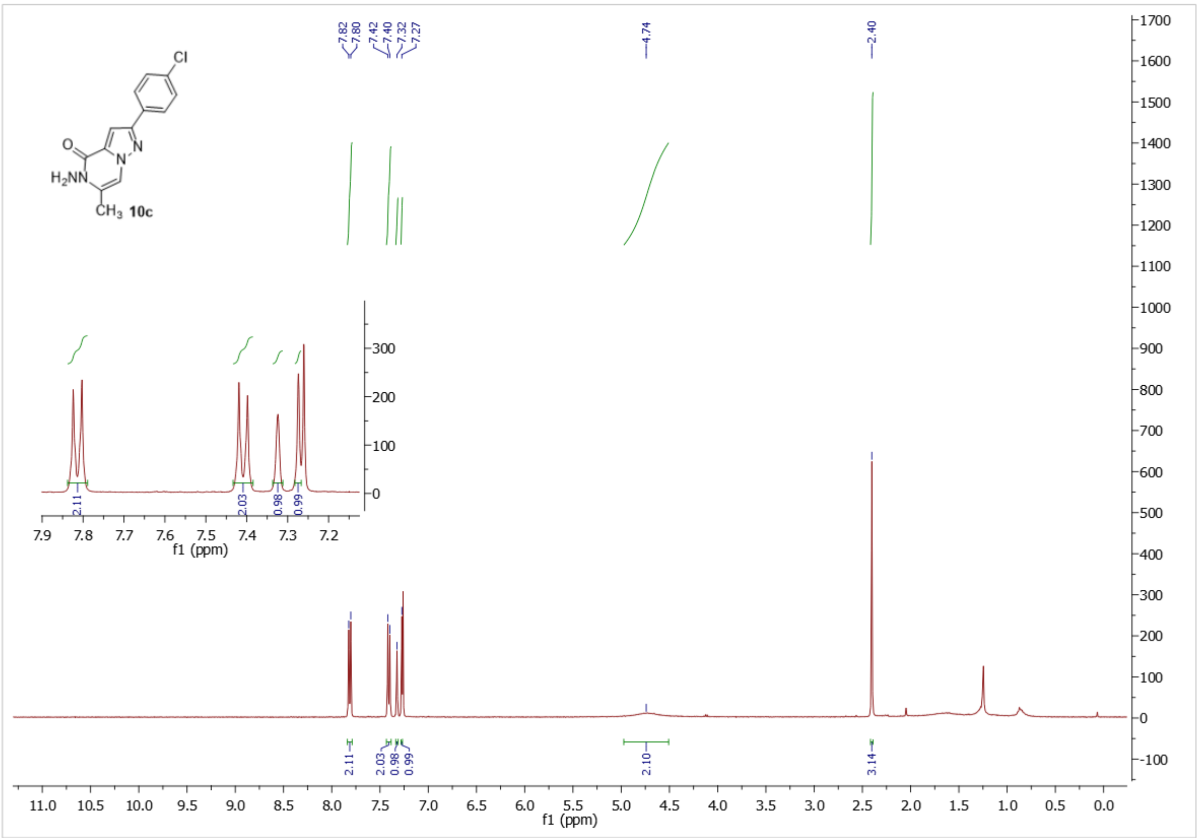

Figure S9. ^{1}H NMR spectrum of compound **10c**

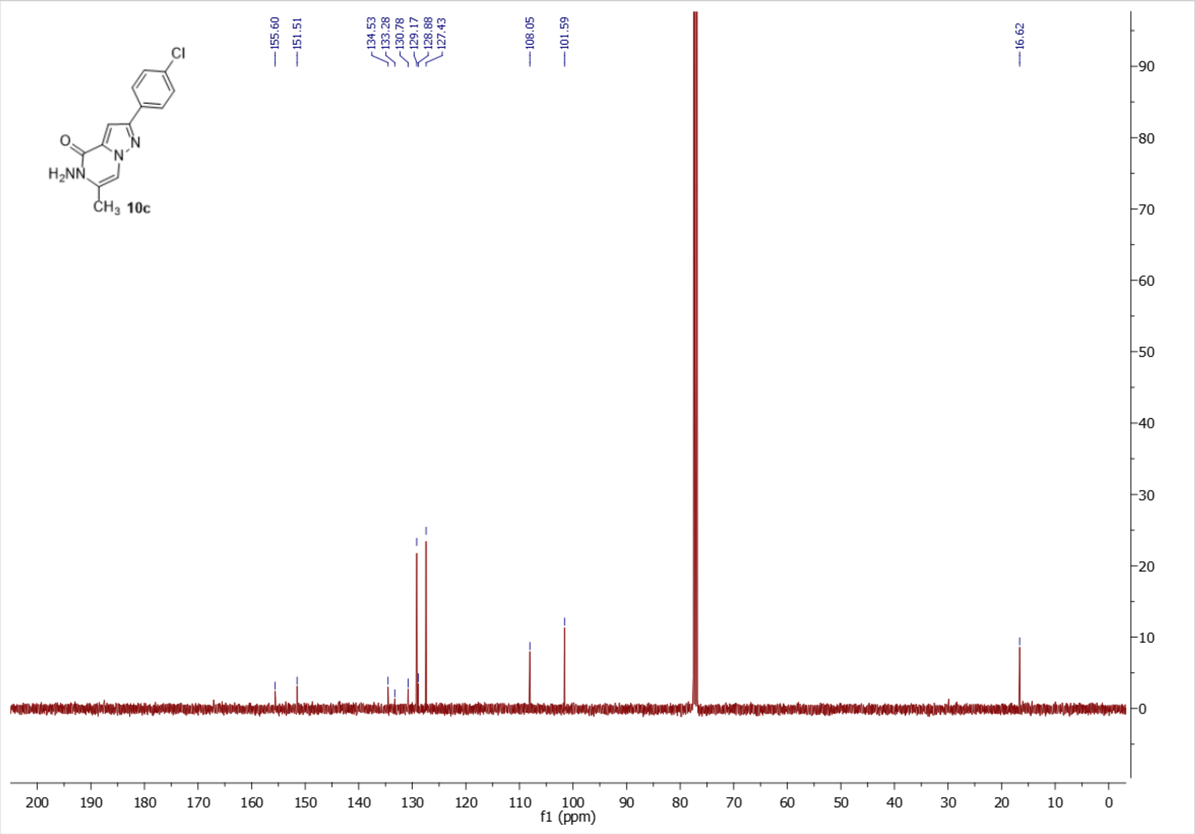

Figure S10. ^{13}C NMR spectrum of compound **10c**

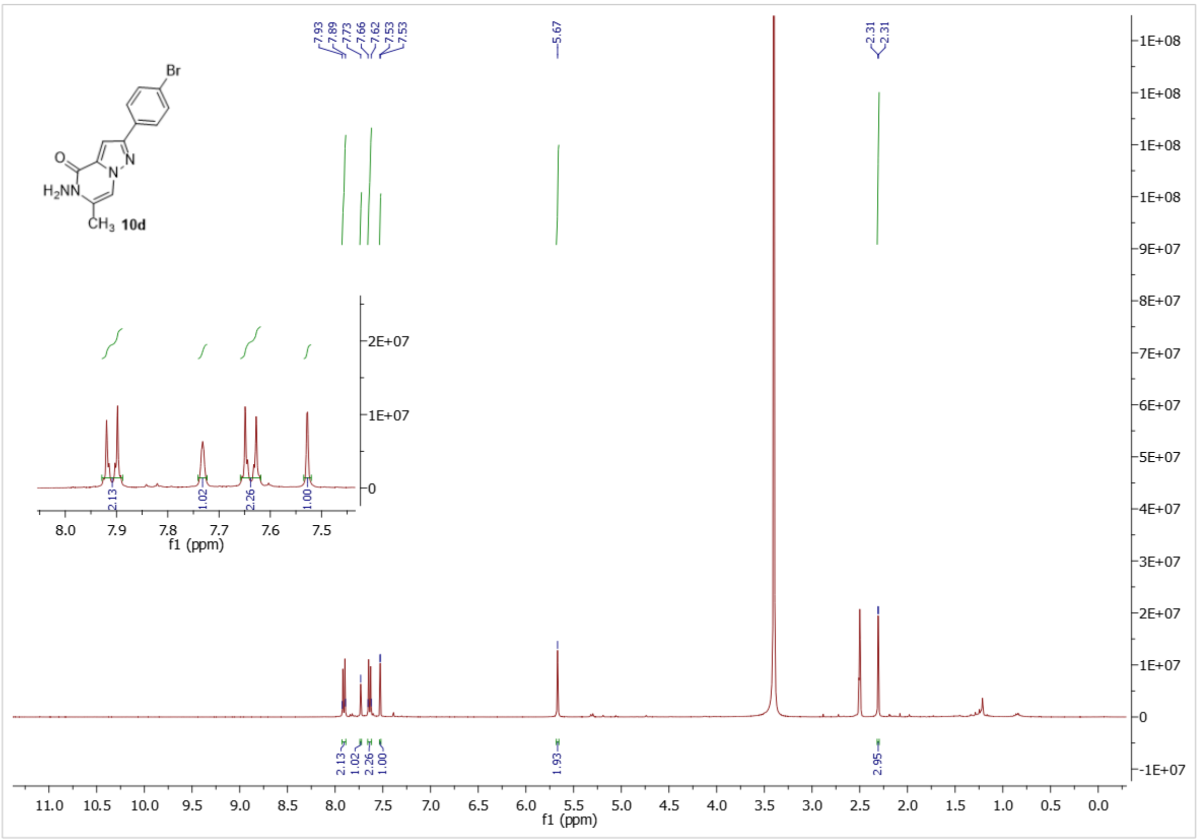

Figure S11. ^{1}H NMR spectrum of compound **10d**

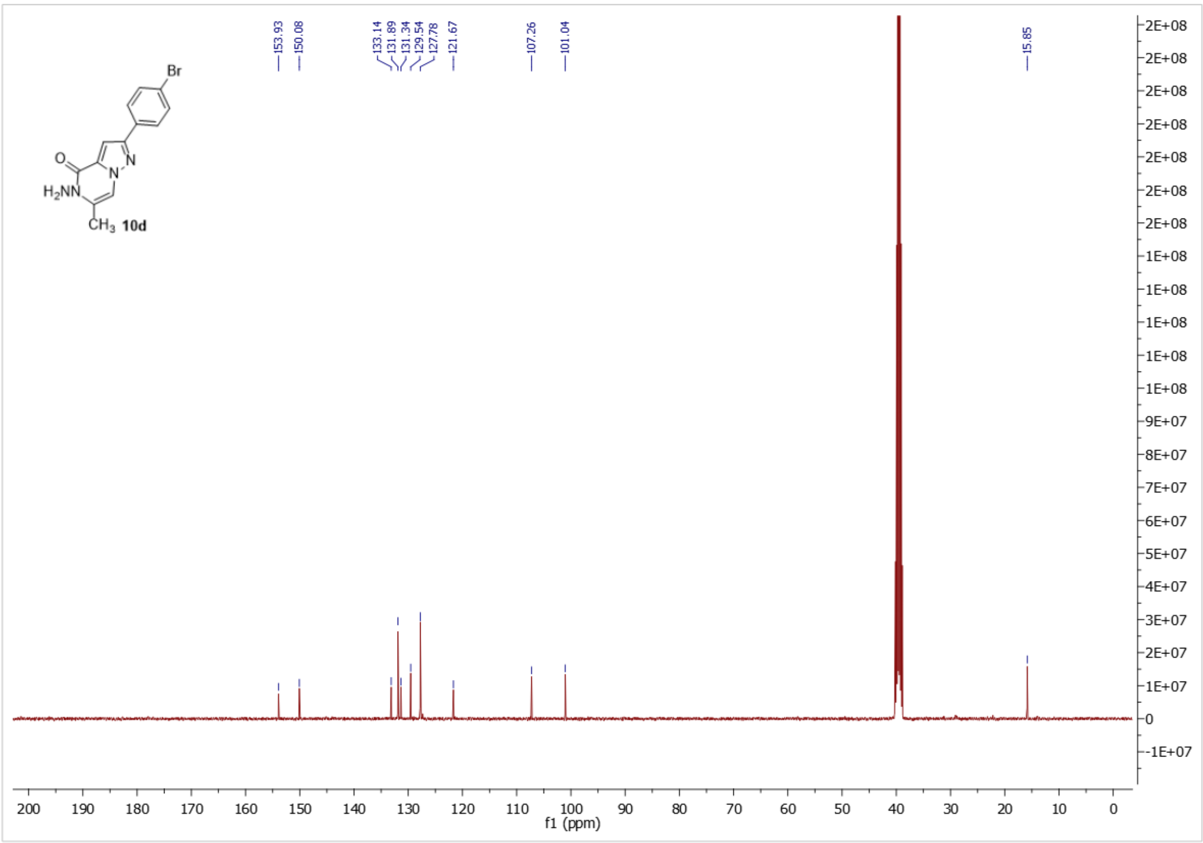

Figure S12. ^{13}C NMR spectrum of compound **10d**

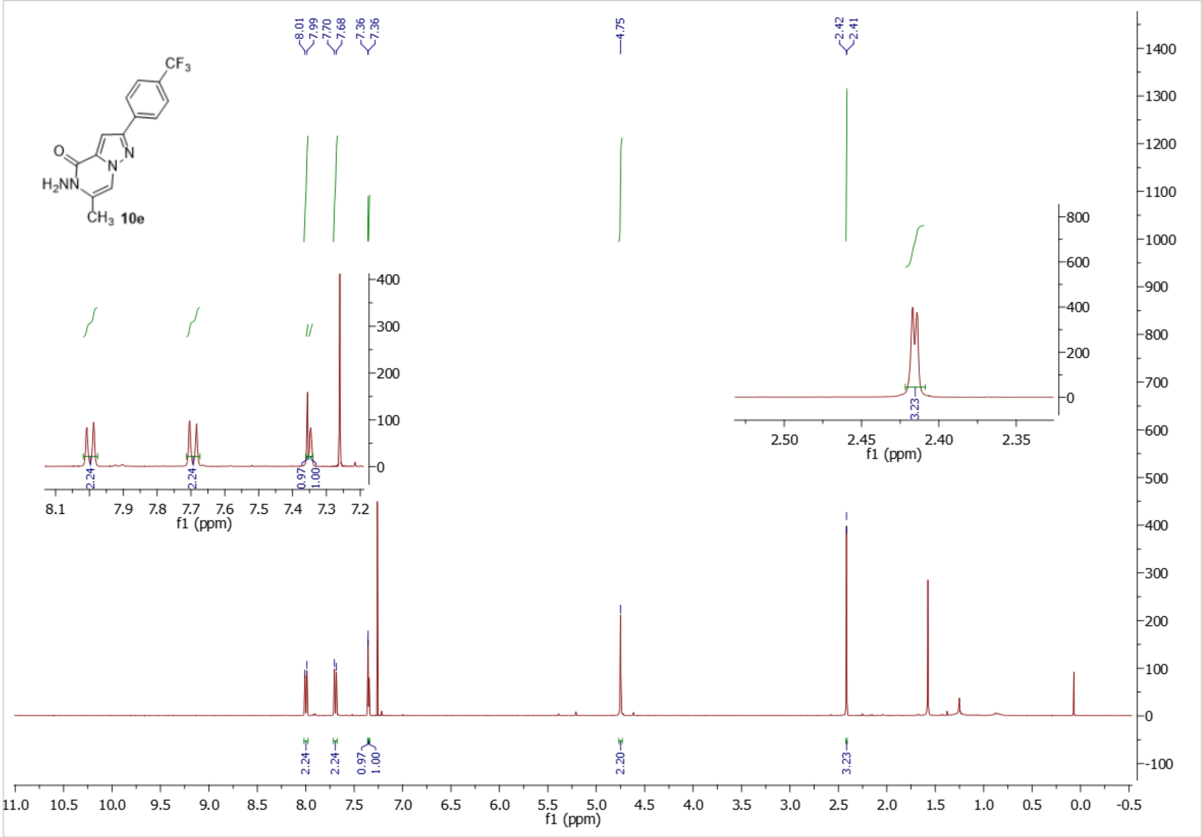

Figure S13. ^{1}H NMR spectrum of compound **10e**

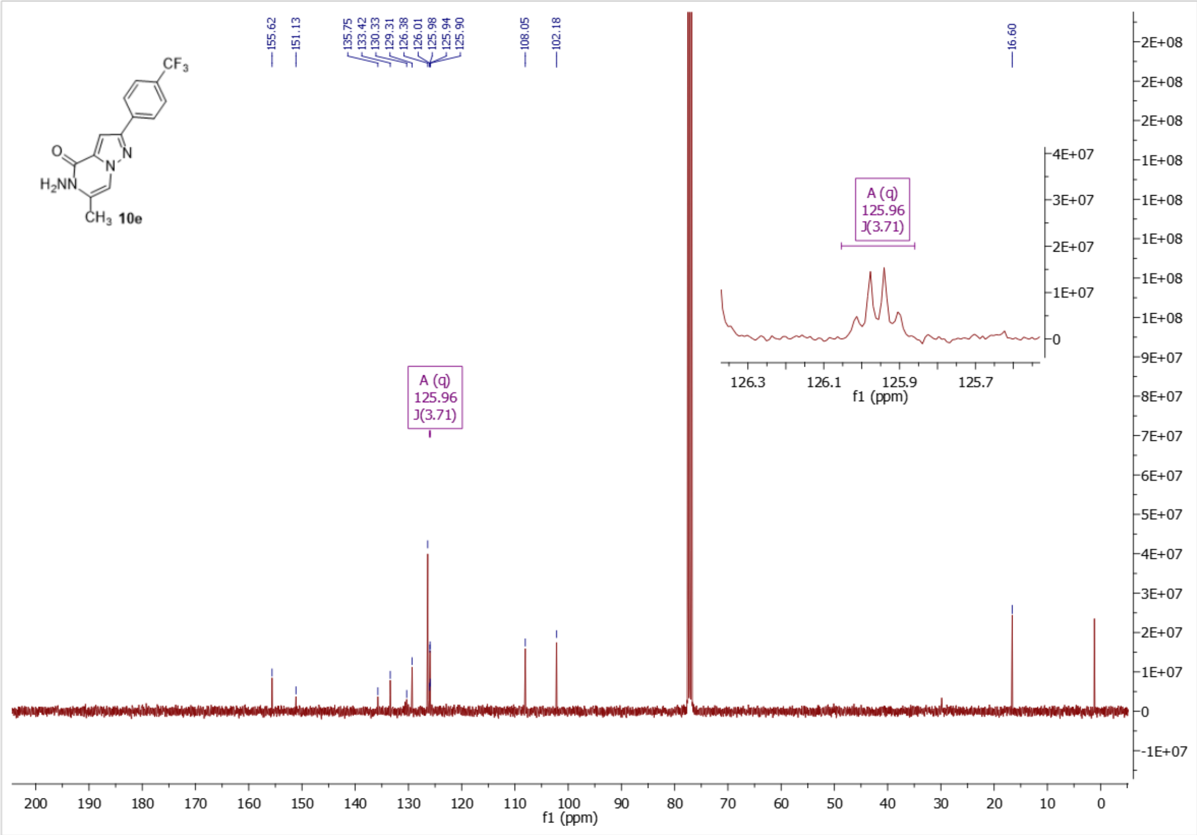

Figure S14. ^{13}C NMR spectrum of compound **10e**

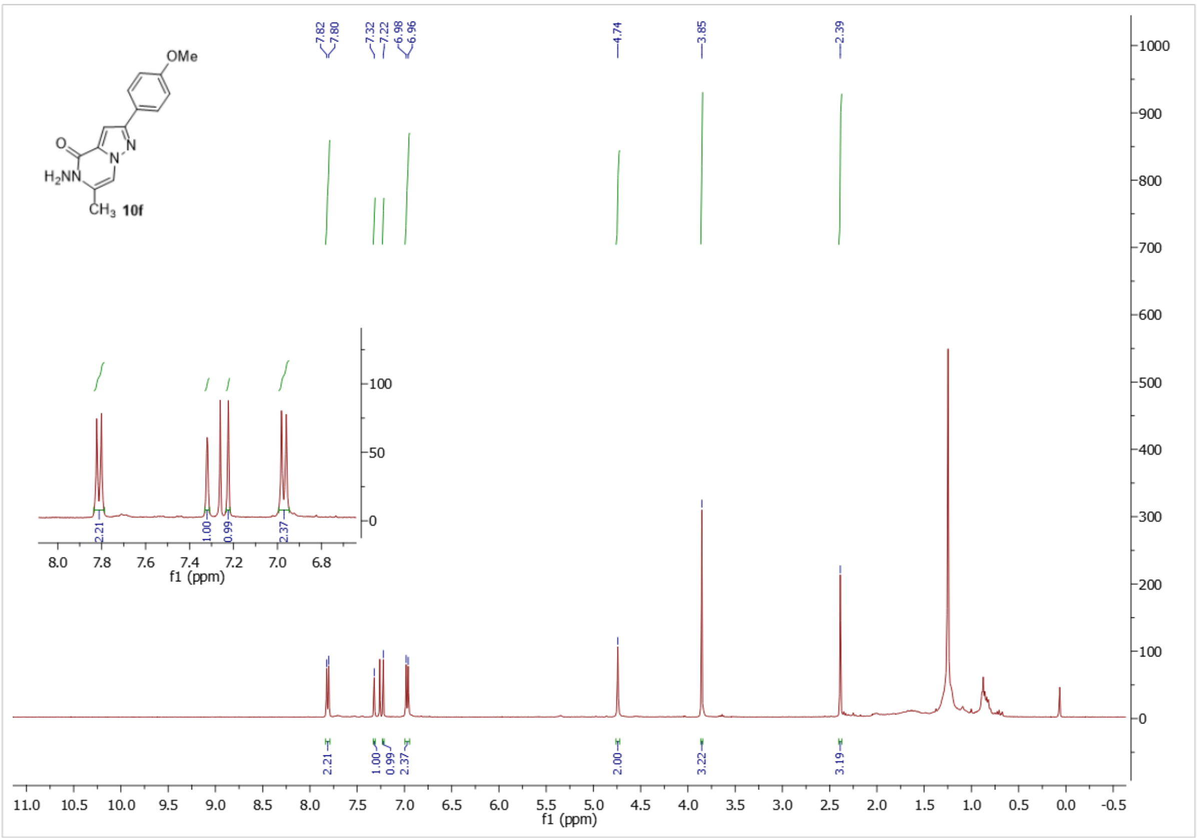

Figure S15. ^{1}H NMR spectrum of compound **10f**

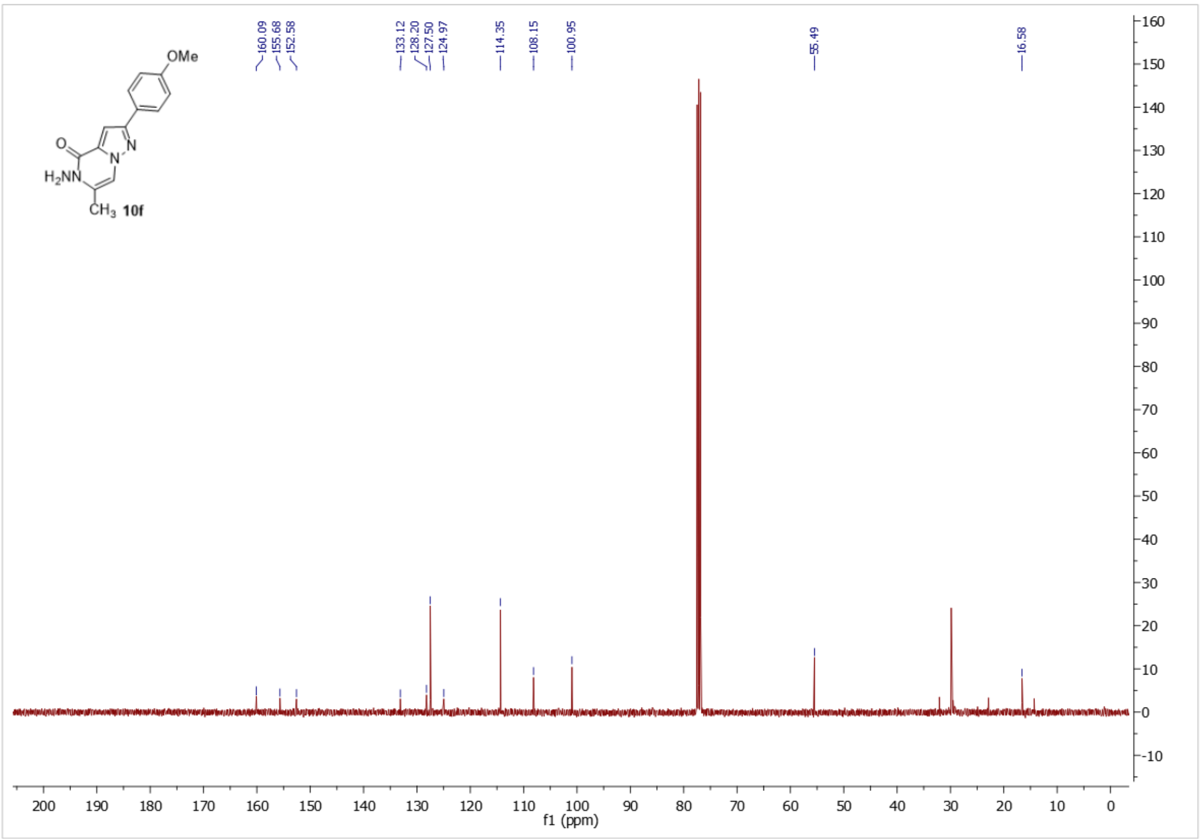

Figure S16. ^{13}C NMR spectrum of compound **10f**

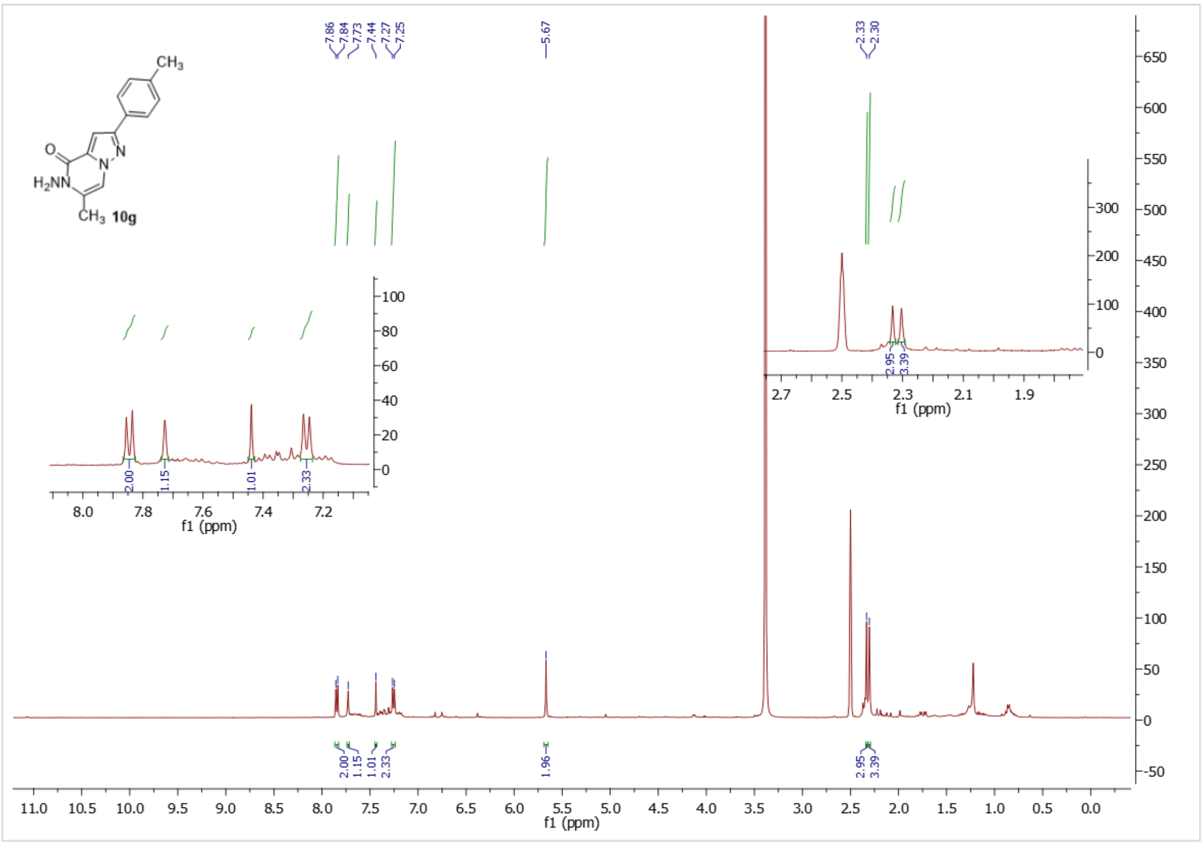

Figure S17. ^{1}H NMR spectrum of compound **10g**

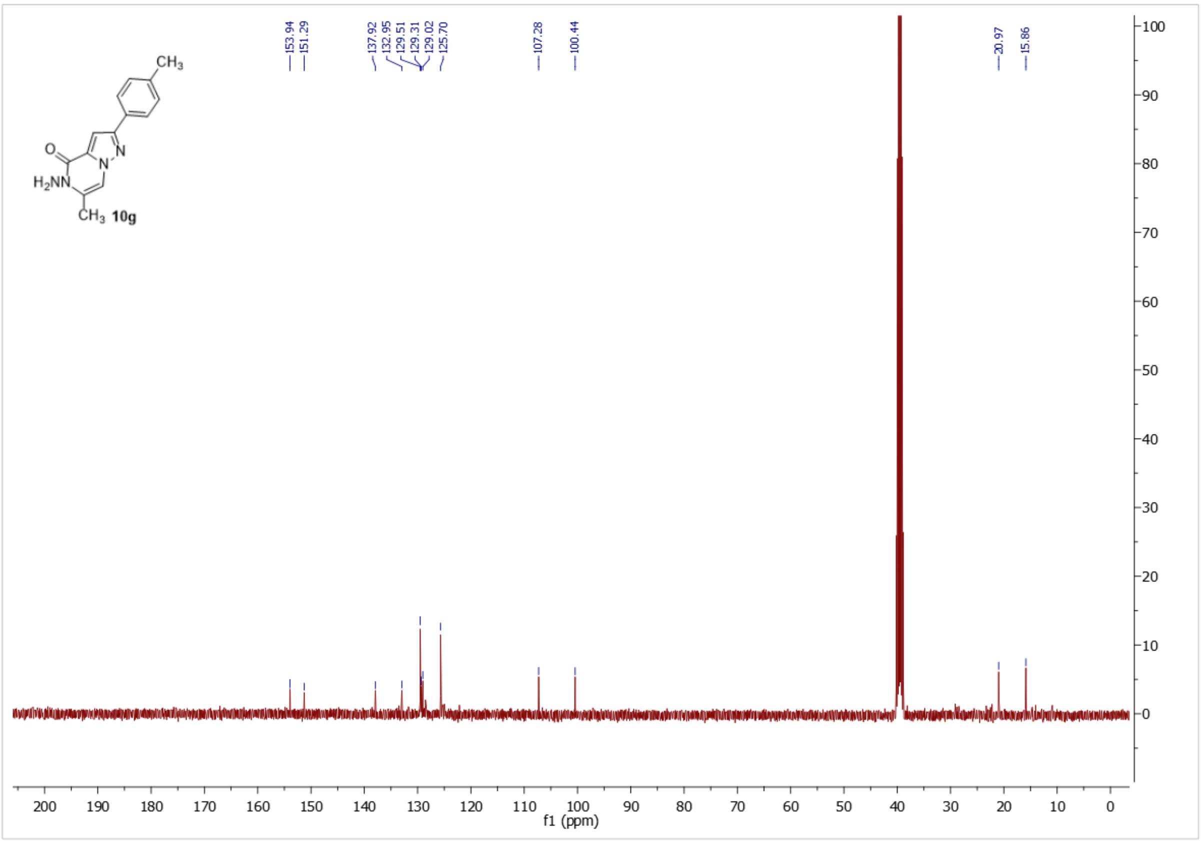

Figure S18. ^{13}C NMR spectrum of compound **10g**

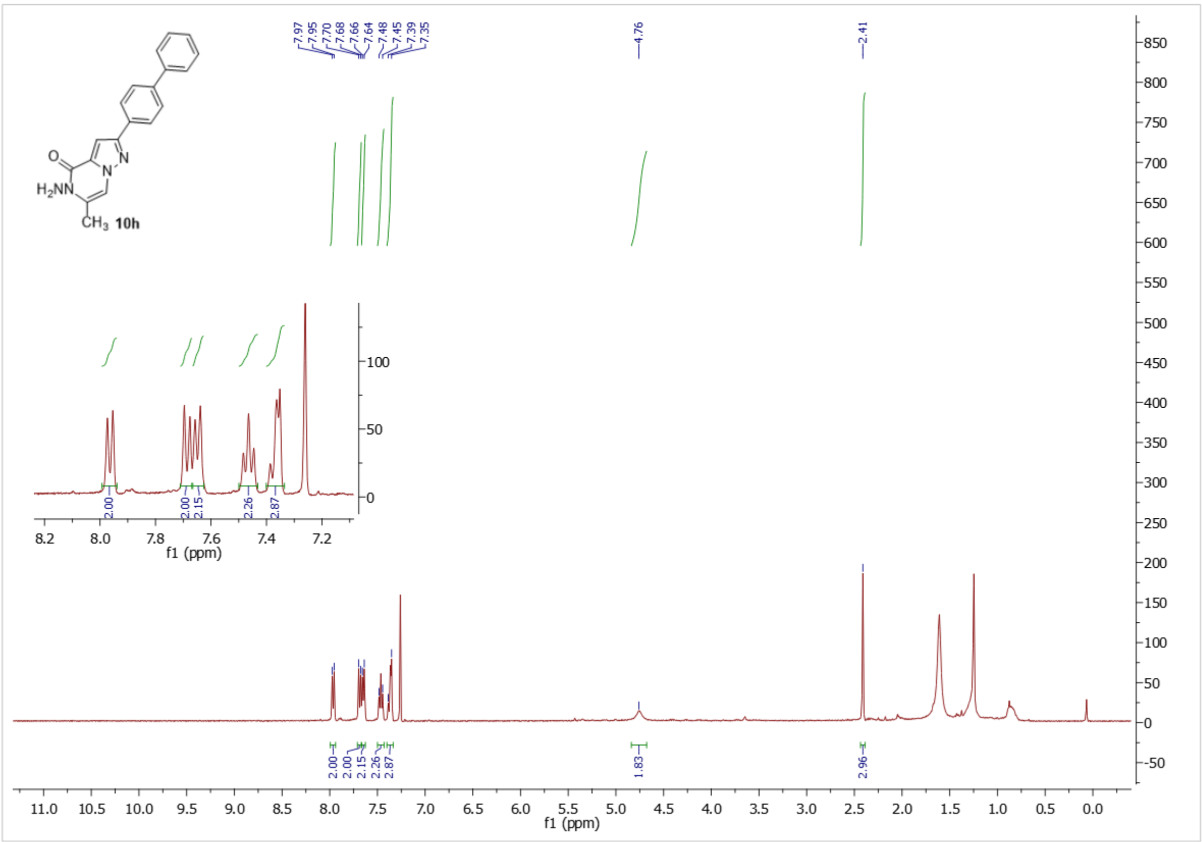

Figure S19. ^{1}H NMR spectrum of compound **10h**

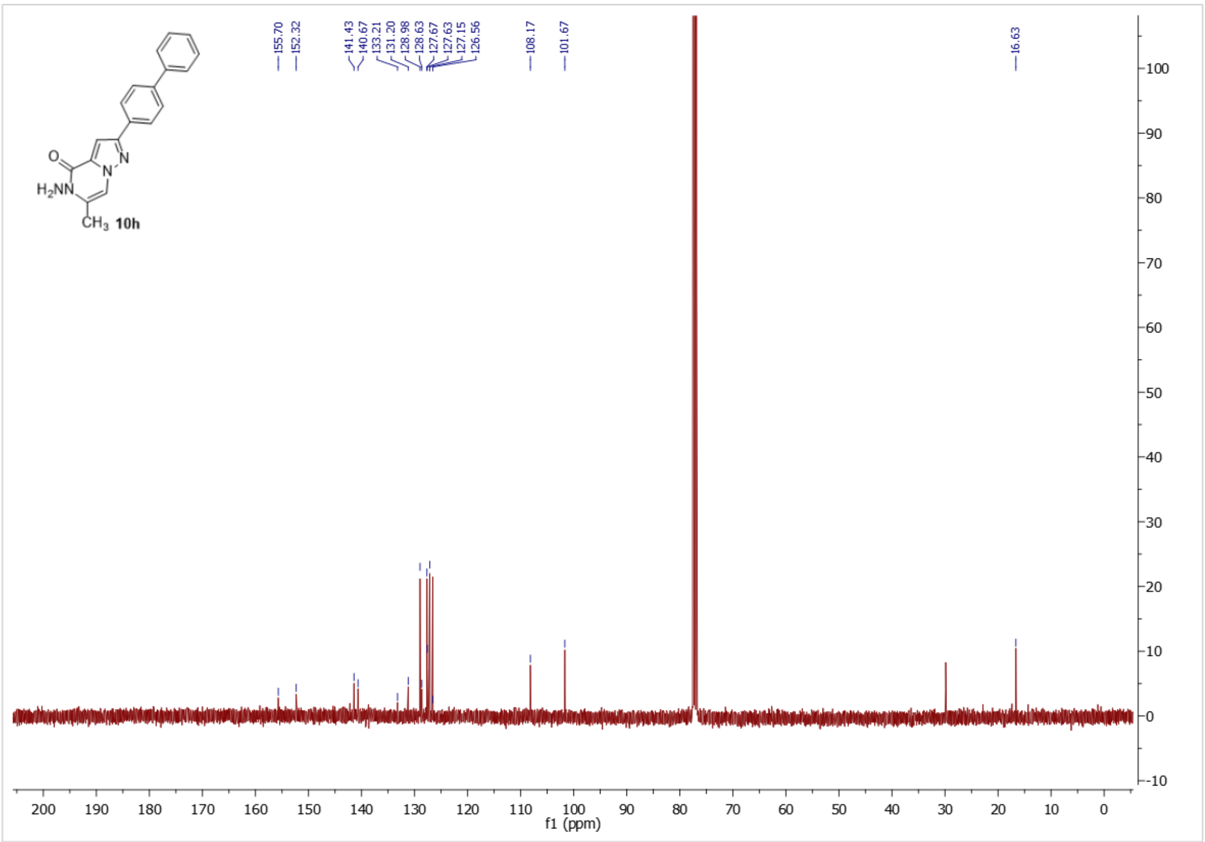

Figure S20. ^{13}C NMR spectrum of compound **10h**

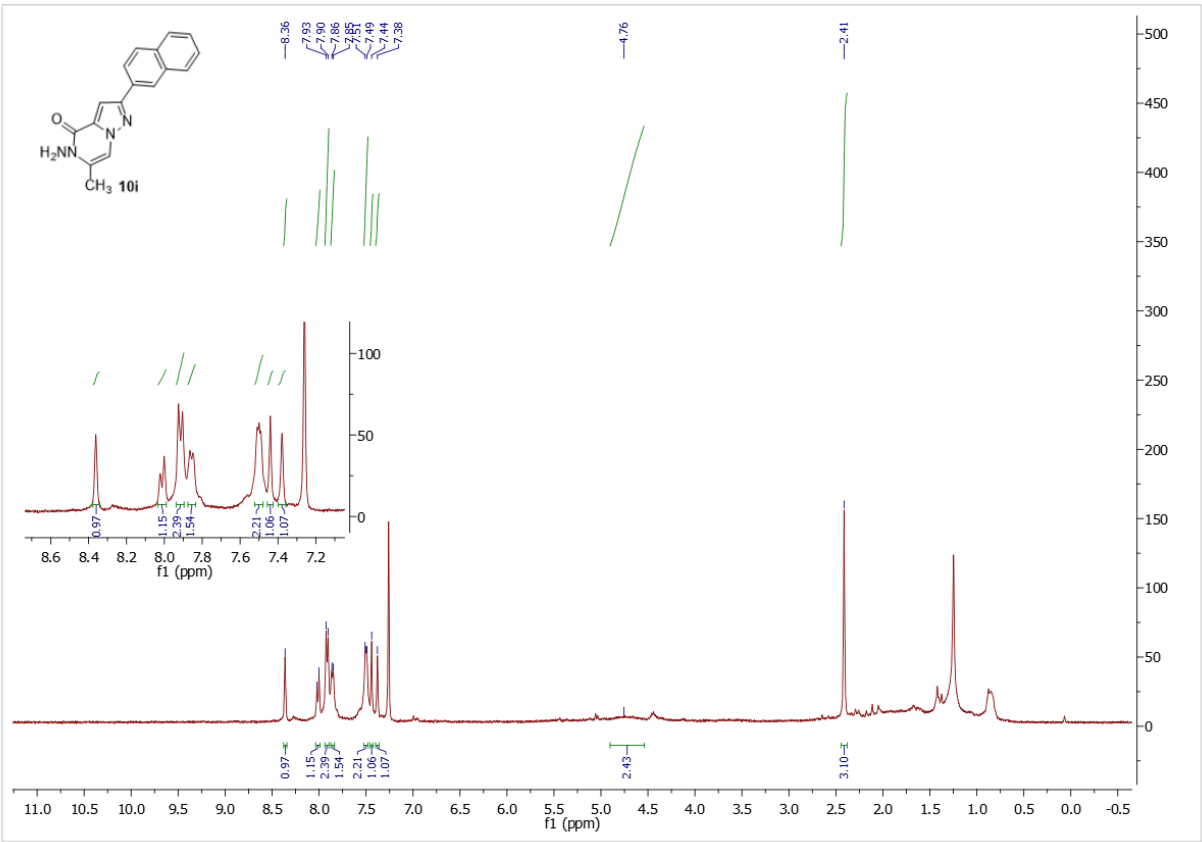

Figure S21. ^{1}H NMR spectrum of compound **10i**

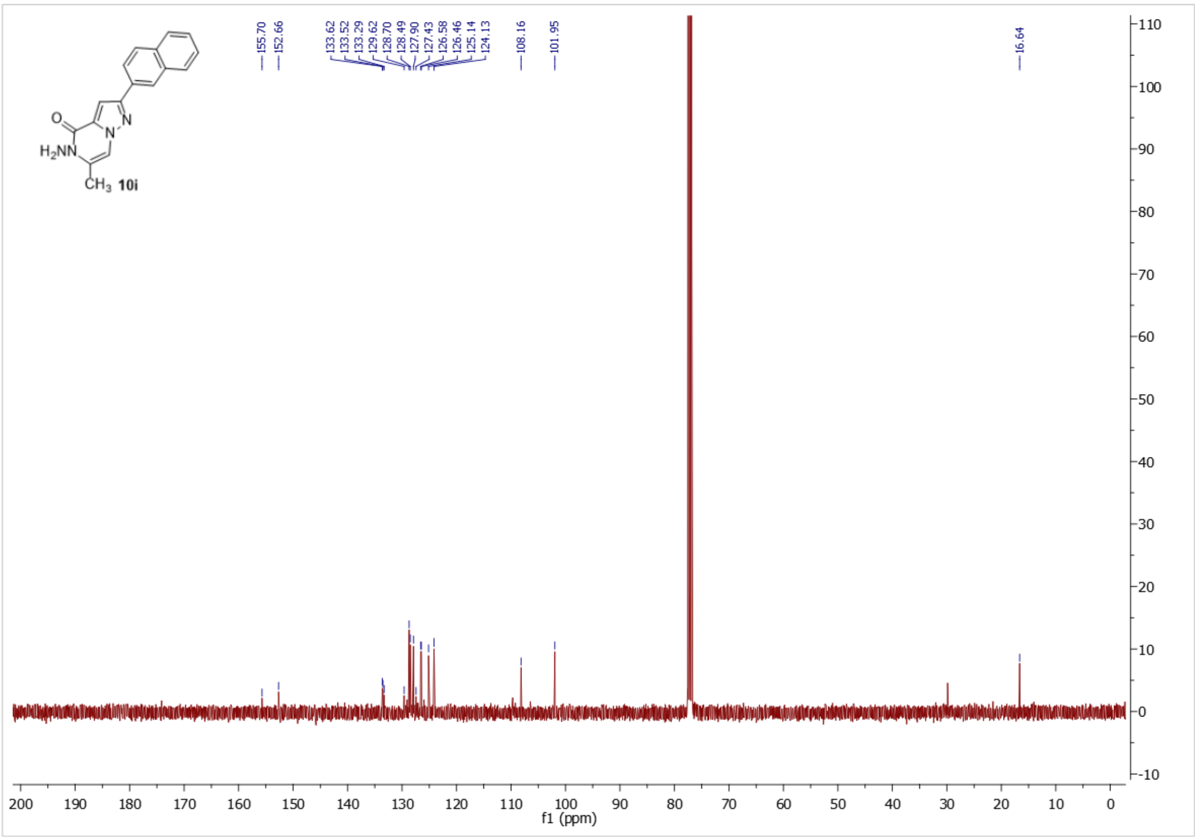

Figure S22. ^{13}C NMR spectrum of compound **10i**

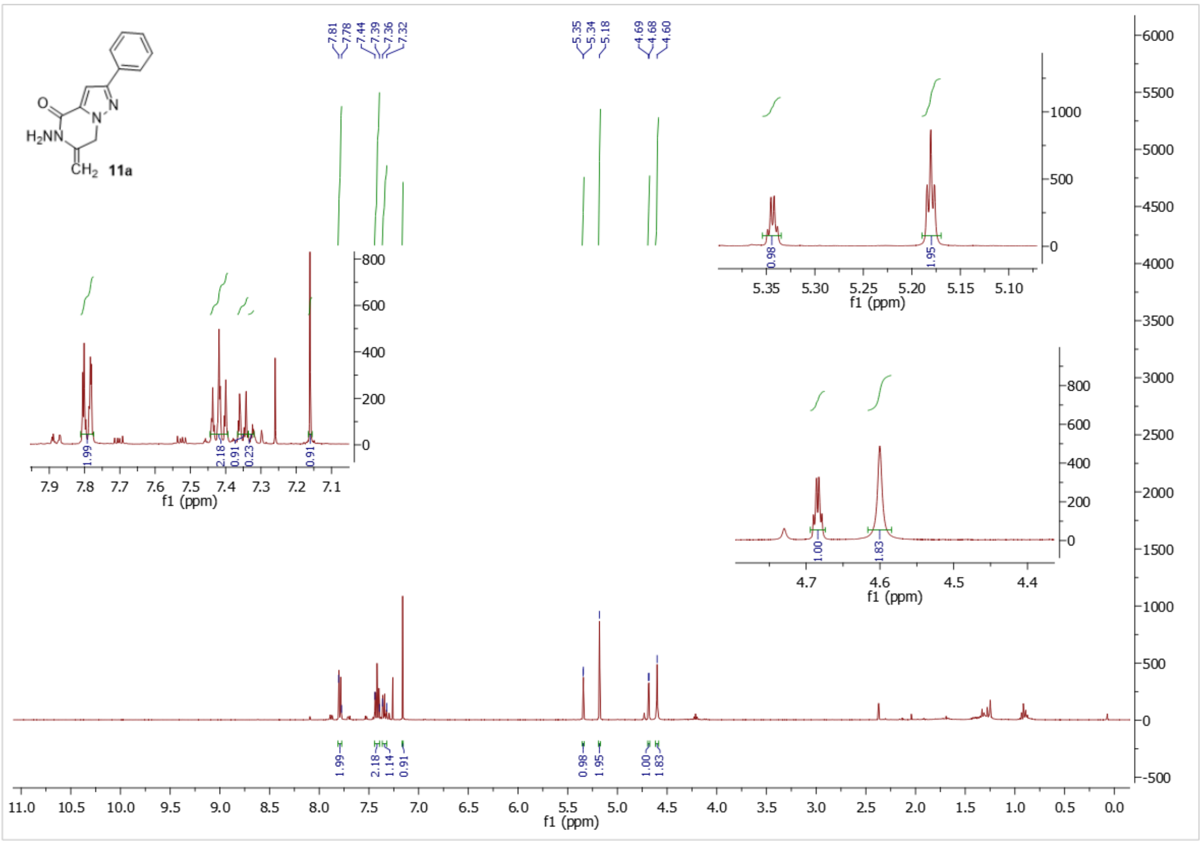

Figure S23. ^{1}H NMR spectrum of compound **11a**

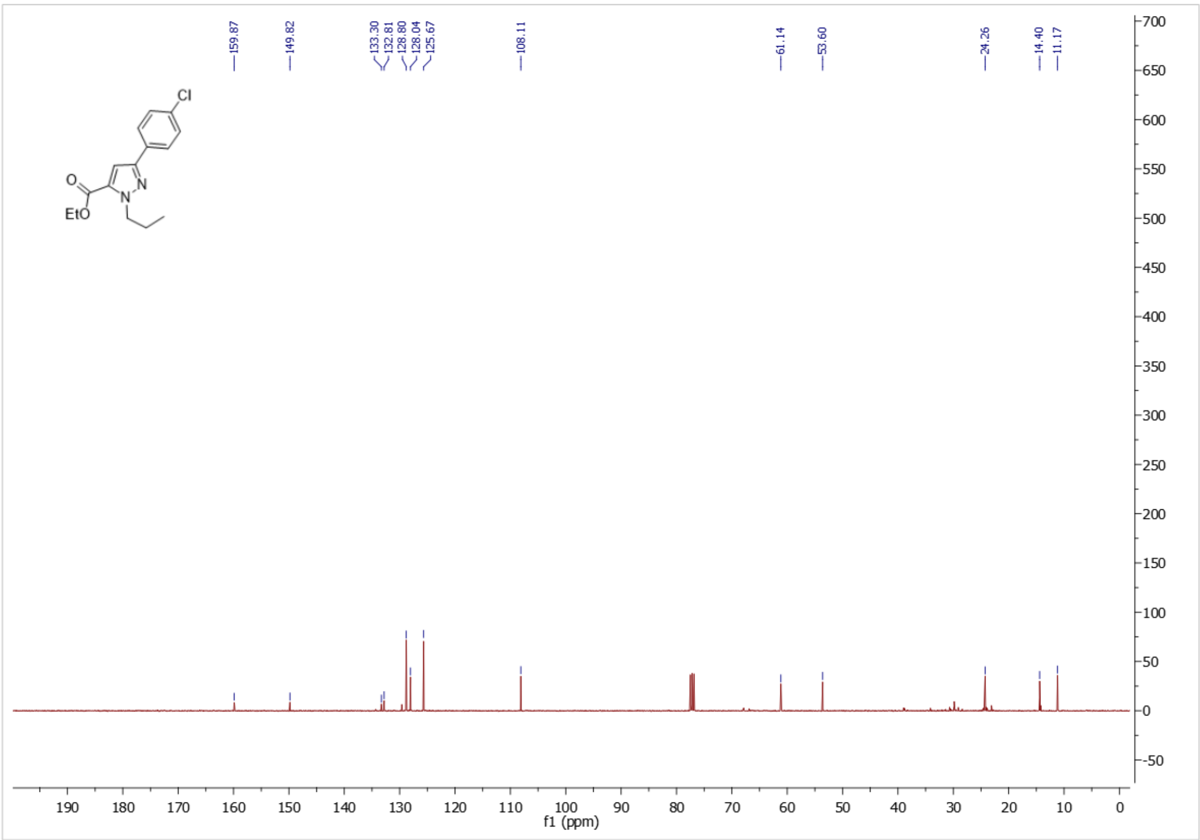

Figure S24. ^{13}C NMR spectrum of compound **12c**

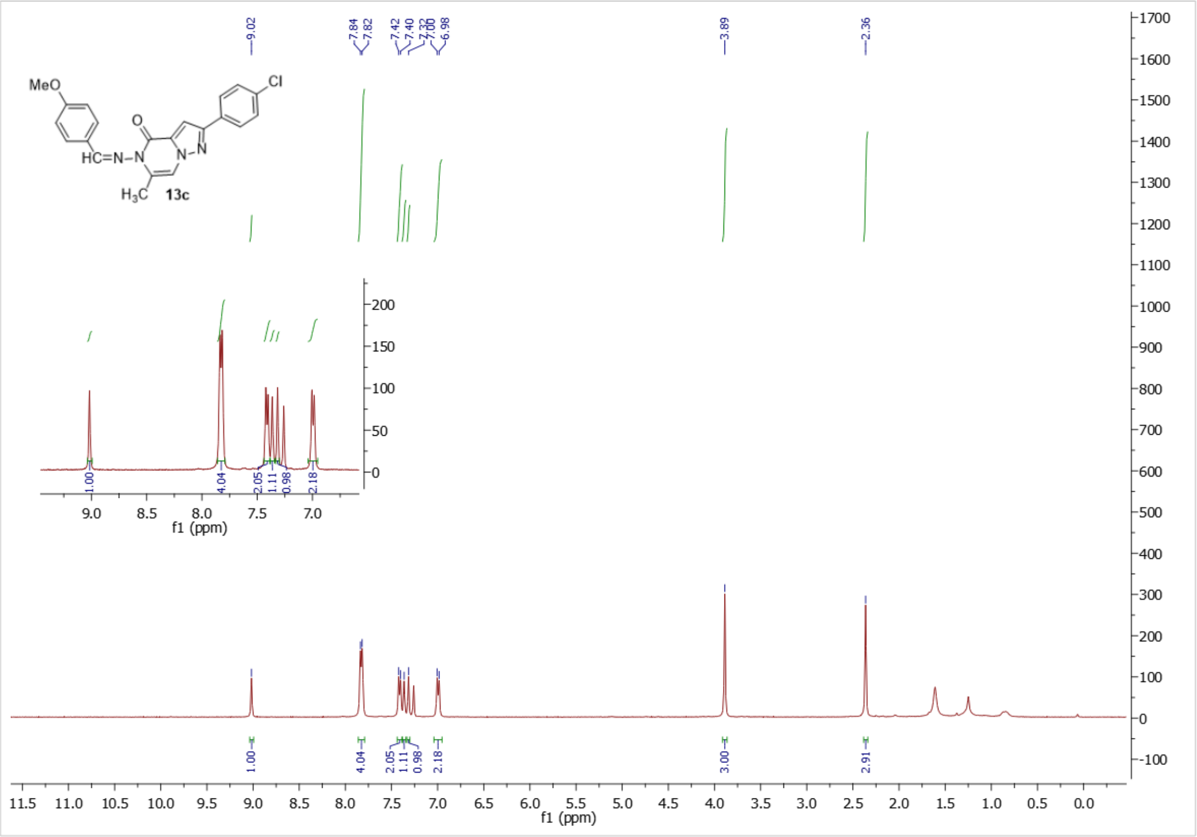

Figure S25. ^{1}H NMR spectrum of compound **13c**

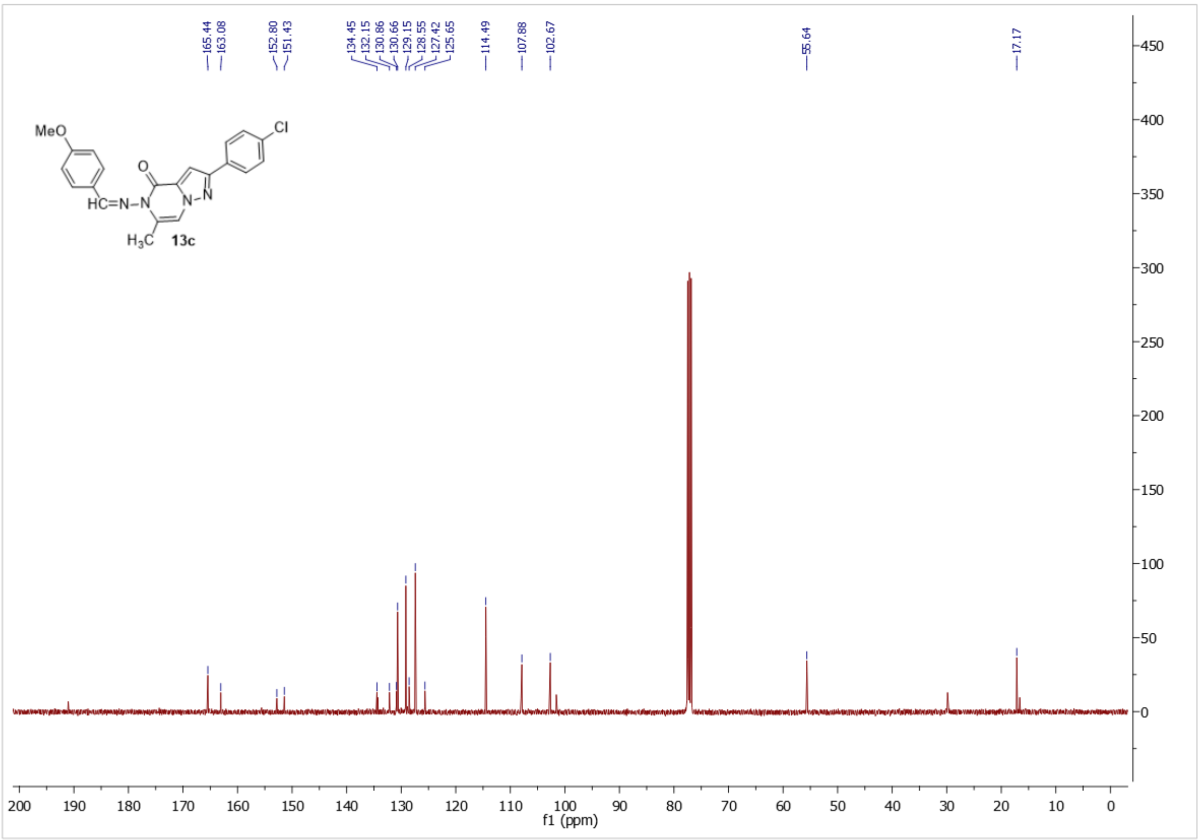

Figure S26. ^{13}C NMR spectrum of compound **13c**

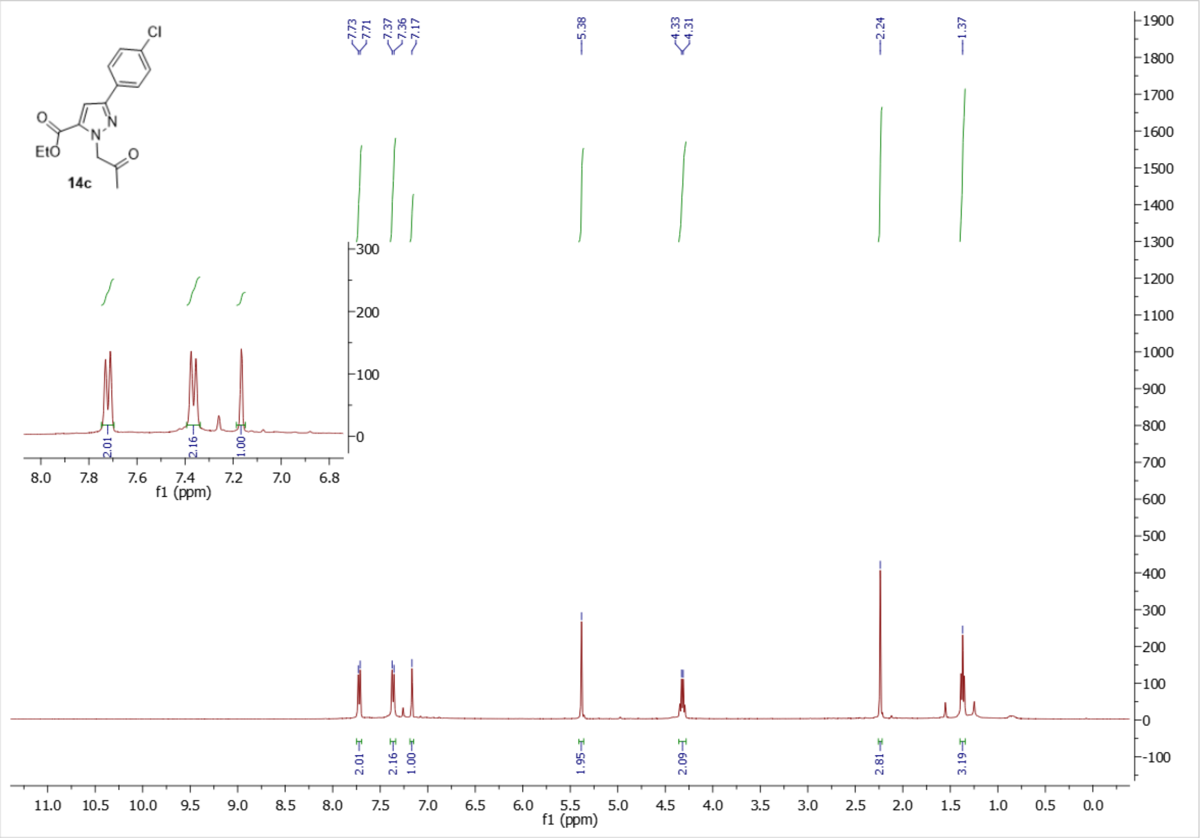

Figure S27. ^{1}H NMR spectrum of compound **14c**

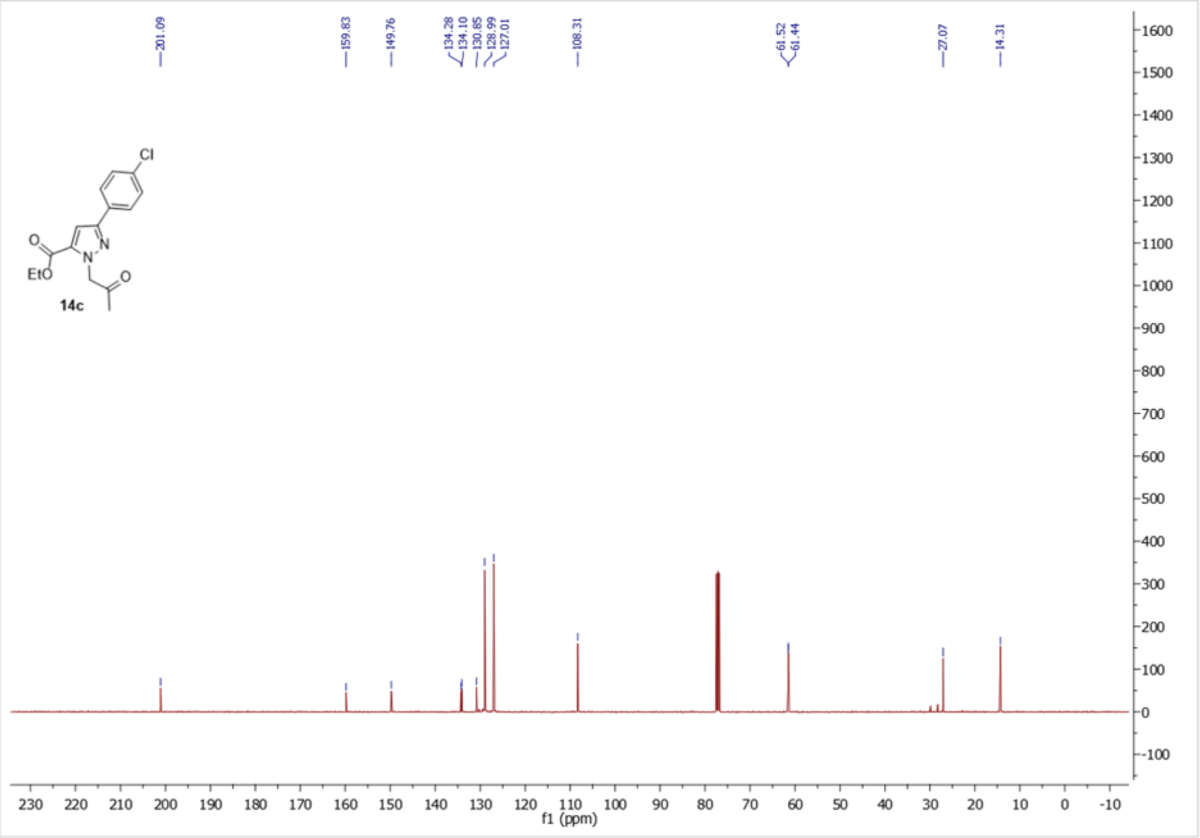

Figure S28. ^{13}C NMR spectrum of compound **14c**

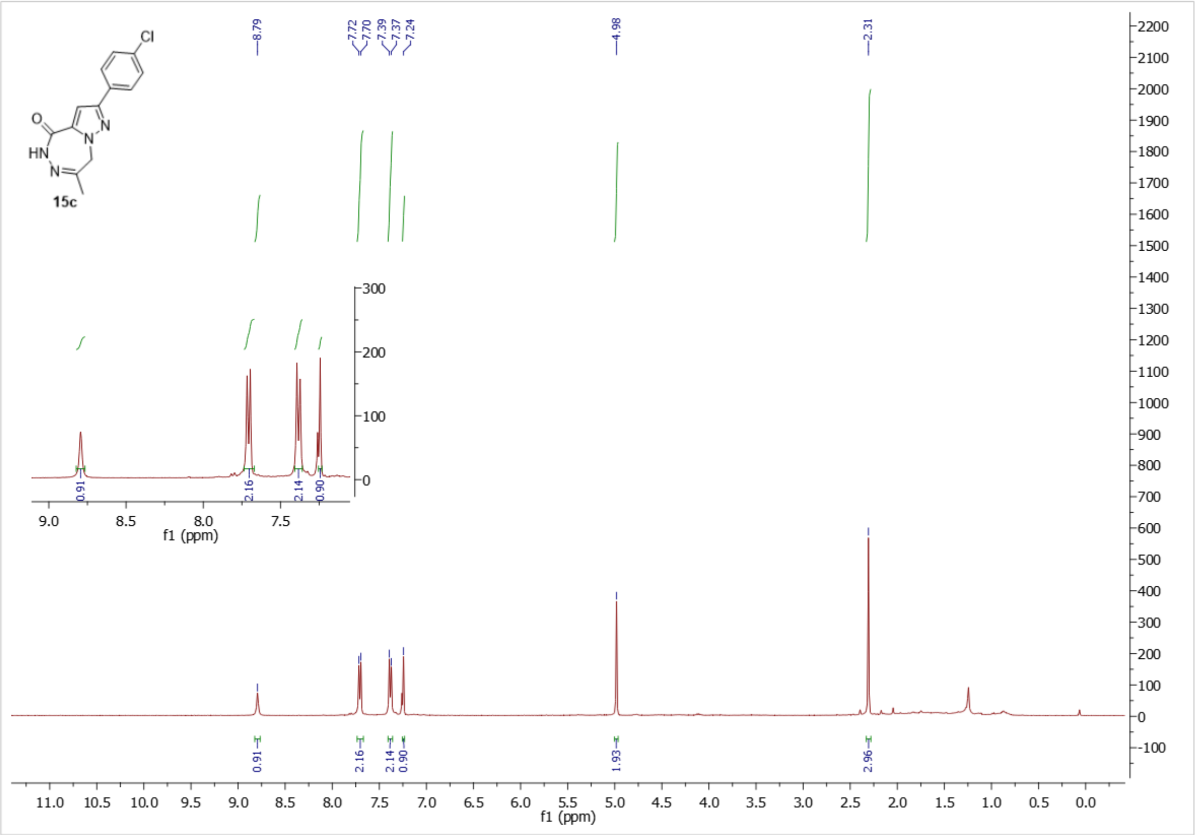

Figure S29. ^{1}H NMR spectrum of compound **15c**

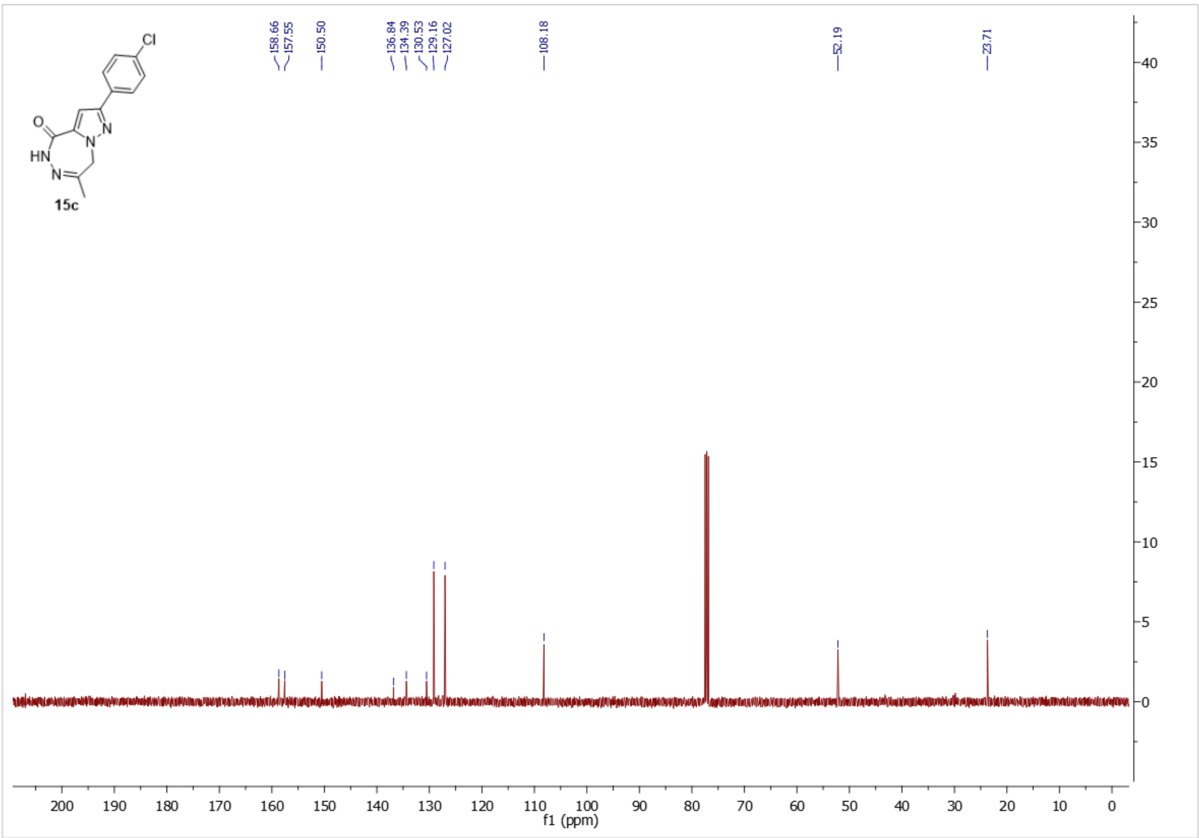

Figure S30. ^{13}C NMR spectrum of compound **15c**

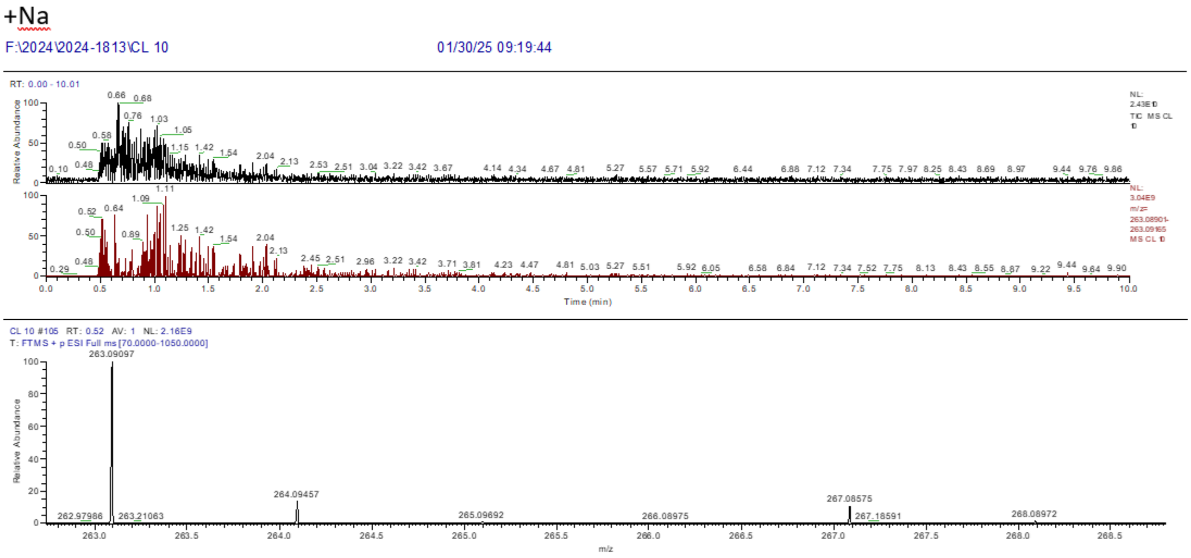

Figure S31. LC-MS/MS spectrum of compound **10a**

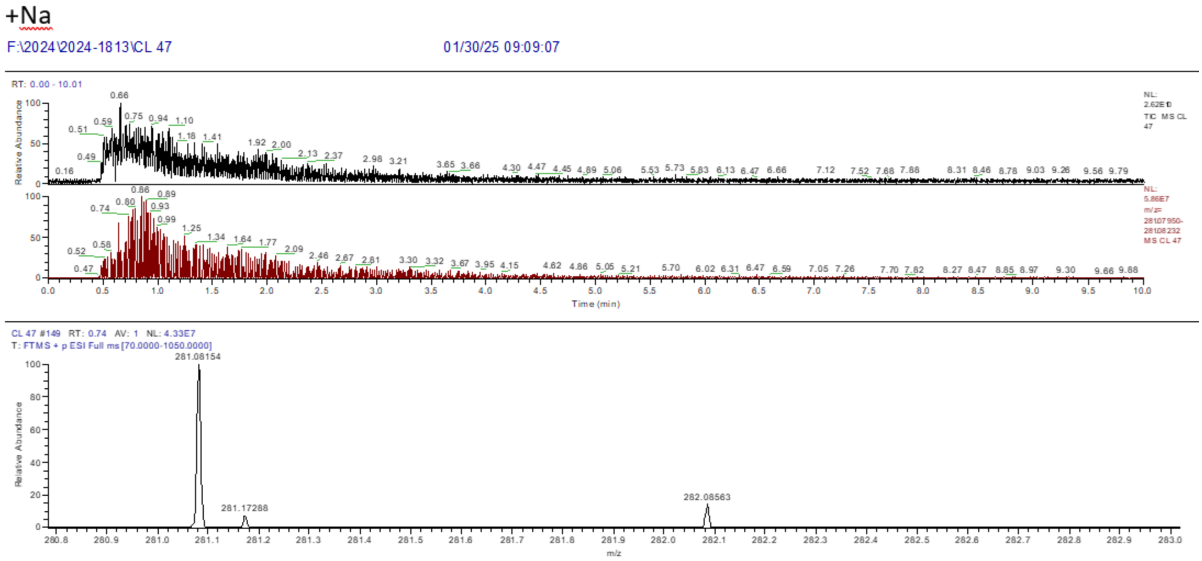

Figure S32. LC-MS/MS spectrum of compound **10b**

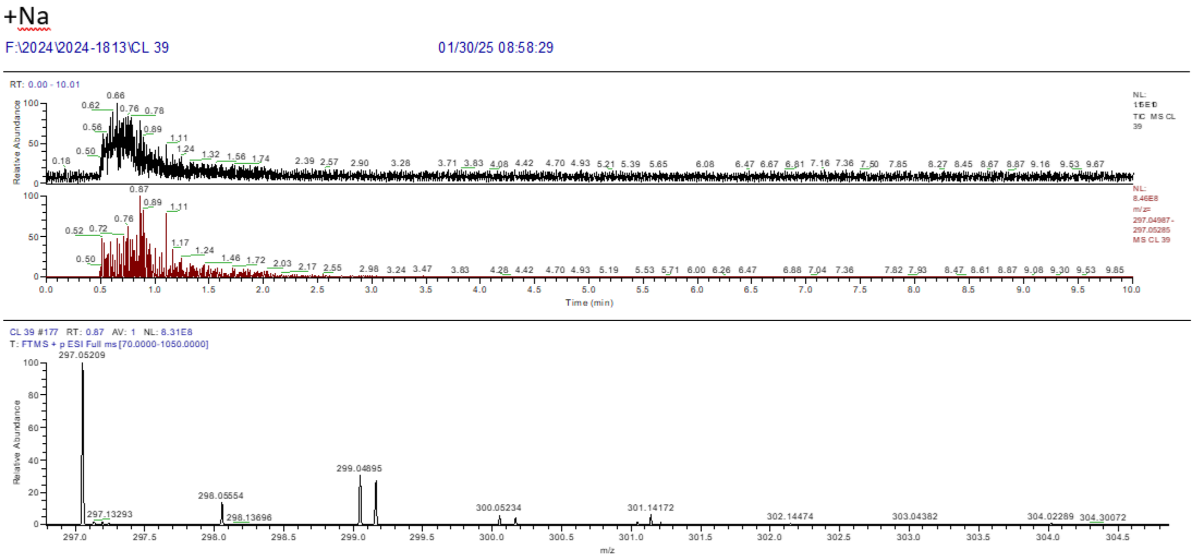

Figure S33. LC-MS/MS spectrum of compound **10c**

H+

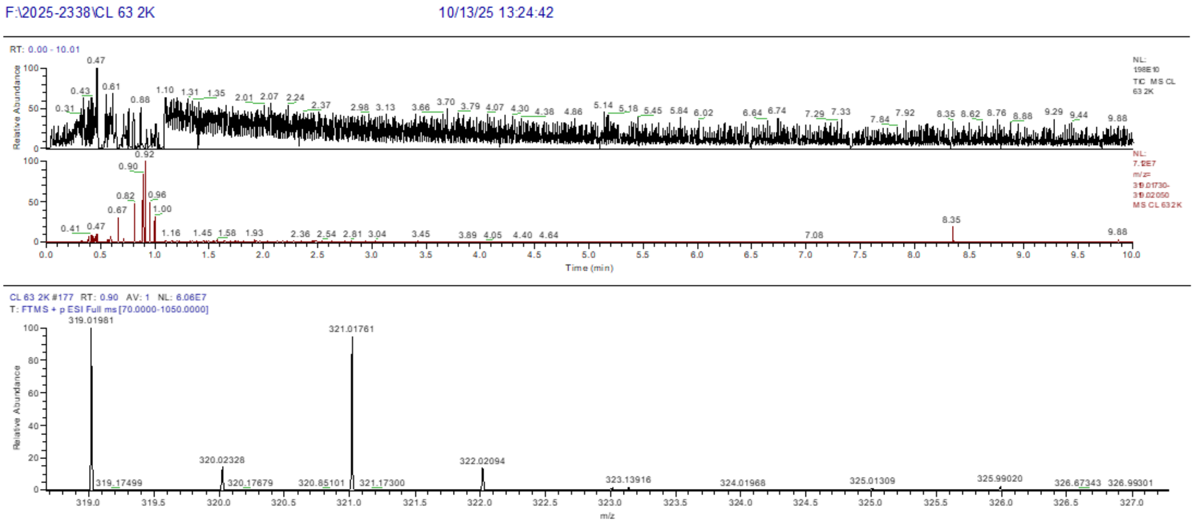

Figure S34. LC-MS/MS spectrum of compound **10d**

+Na

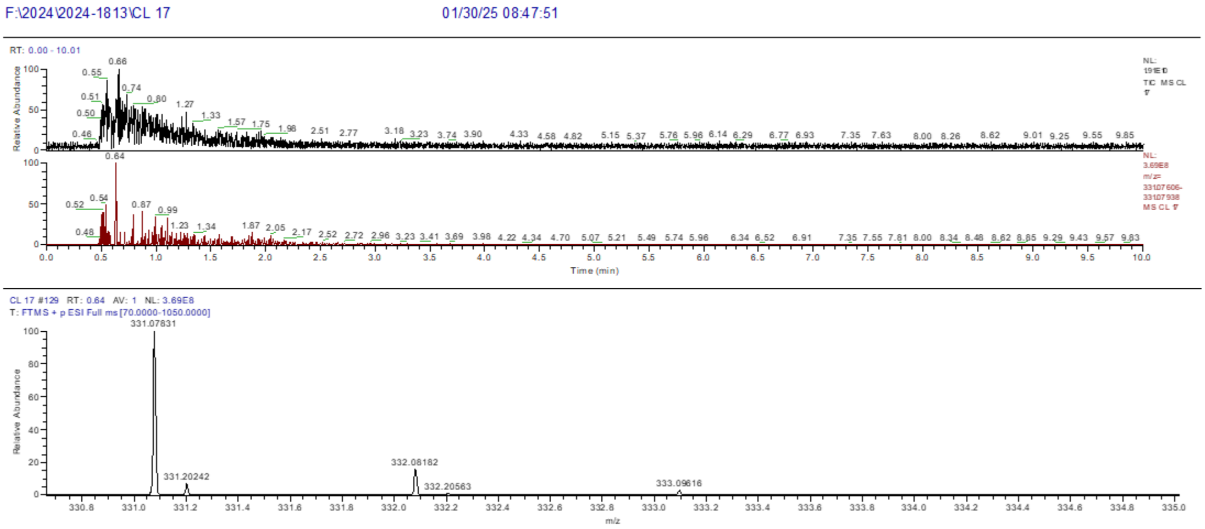

Figure S35. LC-MS/MS spectrum of compound **10e**

+Na

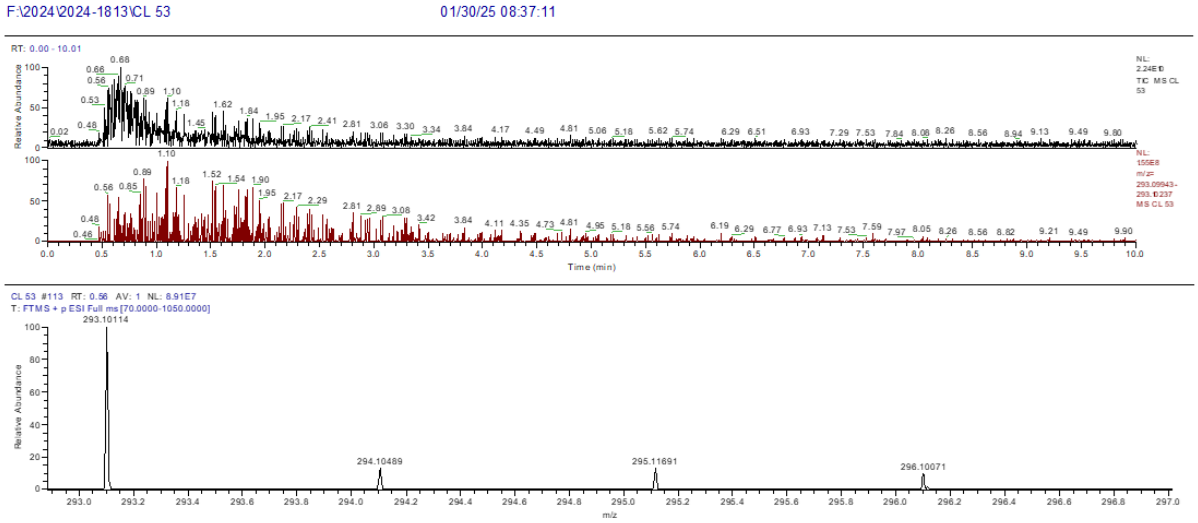

Figure S36. LC-MS/MS spectrum of compound **10f**

H+

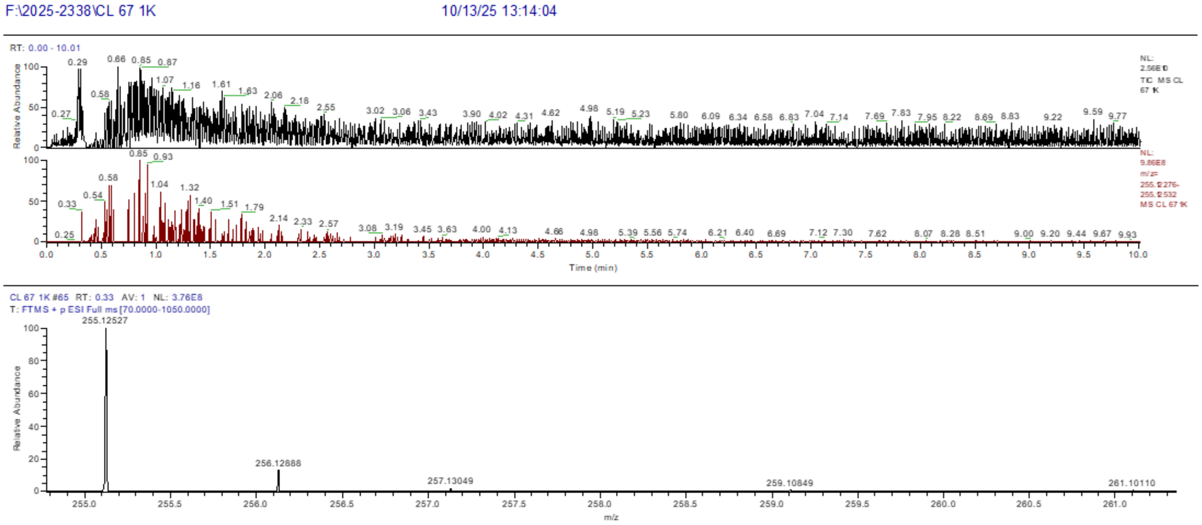

Figure S37. LC-MS/MS spectrum of compound **10g**

H+

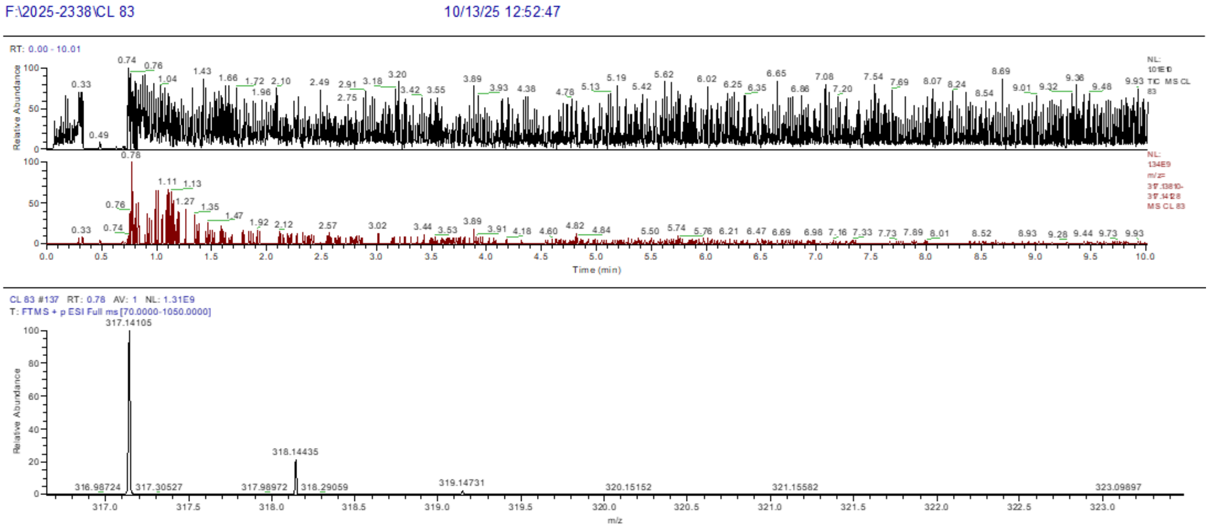

Figure S38. LC-MS/MS spectrum of compound **10h**

H+

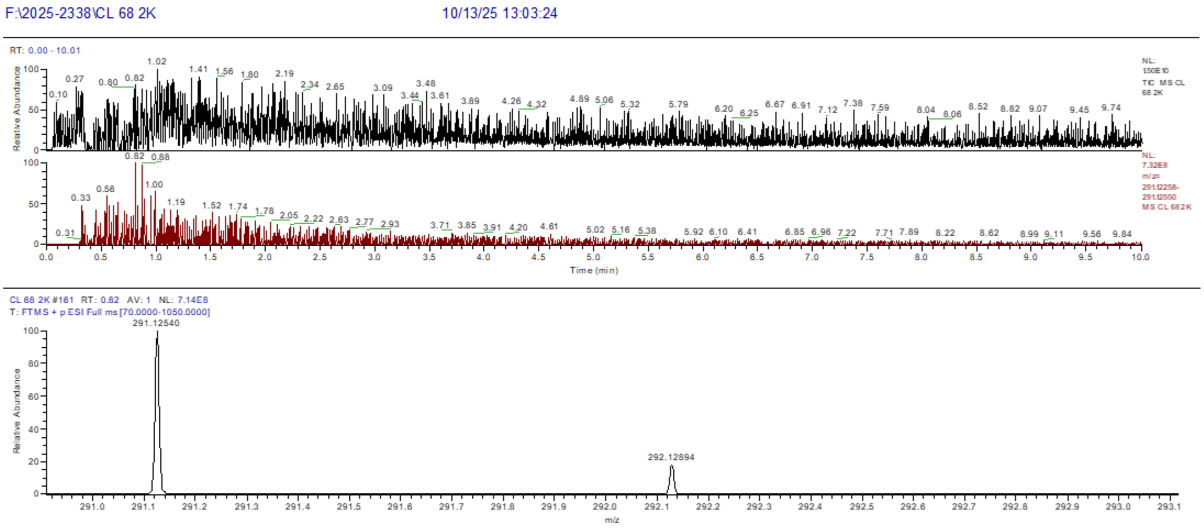

Figure S39. LC-MS/MS spectrum of compound **10i**

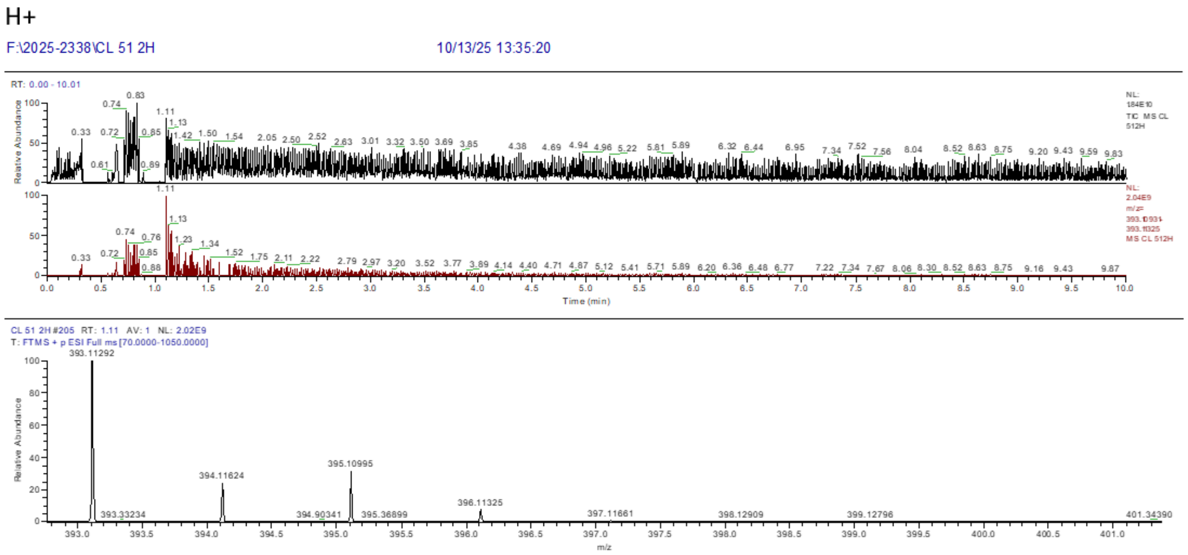

Figure S40. LC-MS/MS spectrum of compound **13c**

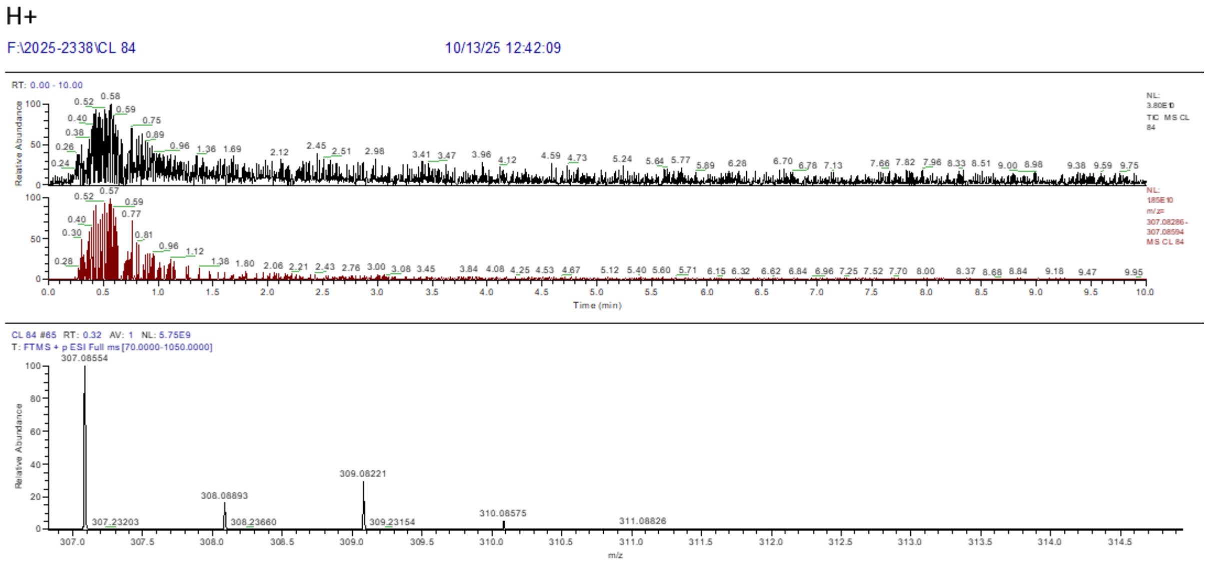

Figure S41. LC-MS/MS spectrum of compound **14c**

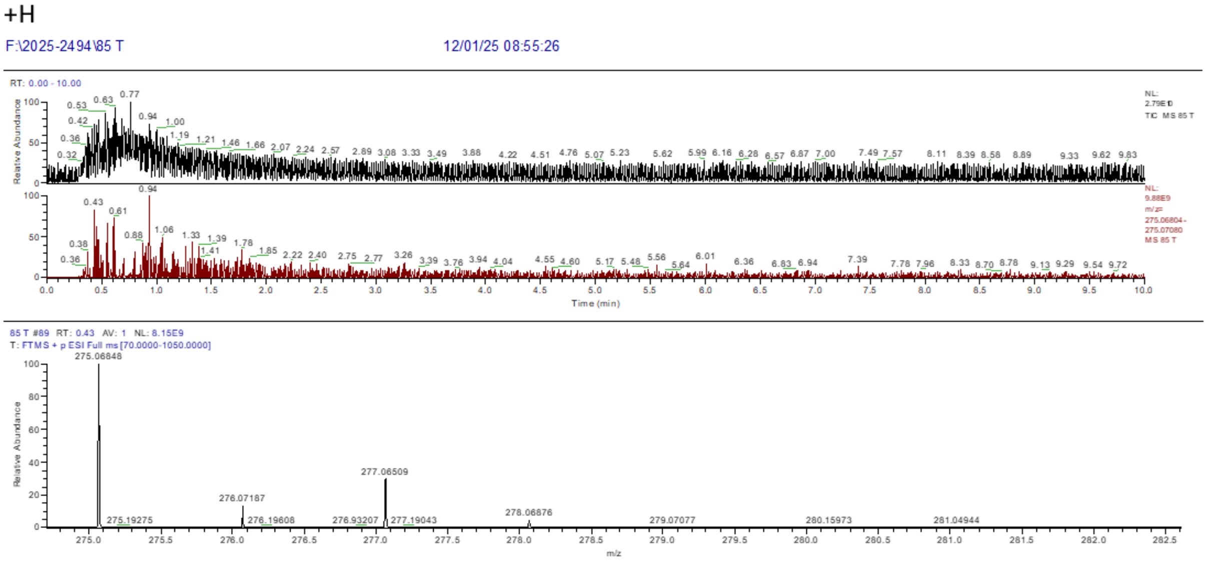

Figure S42. LC-MS/MS spectrum of compound **15c**
